# Supplementary material for: Deciphering the atomistic mechanism underlying highly tunable piezoelectric properties in perovskite ferroelectrics via transition metal doping
Source: Nat Commun. 2024 Dec 5;15:10619. doi: 10.1038/s41467-024-54842-6 (PMC11621366; doi:10.1038/s41467-024-54842-6)
Supplement: Supplementary file 1 — Supplementary Information [file 41467_2024_54842_MOESM1_ESM.pdf]

Supplementary Information

Deciphering the Mechanism Underlying Highly Adjustable Piezoelectric Properties in Perovskite Ferroelectrics via Transition Metal Doping

Peng Tan[1,†], Xiaolin Huang[1,†], Yu Wang[1,†], Bohan Xing[1], Jiajie Zhang[2], Chengpeng Hu[1], Xiangda Meng[1], Xiaodong Xu[3], Danyang Li[4], Xianjie Wang[1], Xin Zhou[5], Nan Zhang[2], Qisheng Wang[6], Fei Li[2,*], Shujun Zhang[7,*], Hao Tian[1,*]

*Corresponding author. Email: ful5@xjtu.edu.cn (F.L.); shujun@uow.edu.au (S.Z.); tianhao@ hit.edu.cn (H.T.)

[†]These authors contributed equally to this work.

This PDF file includes:

Figure S1 to Figure S14

Table S1 to Table S7

Appendix S1 to Appendix S6

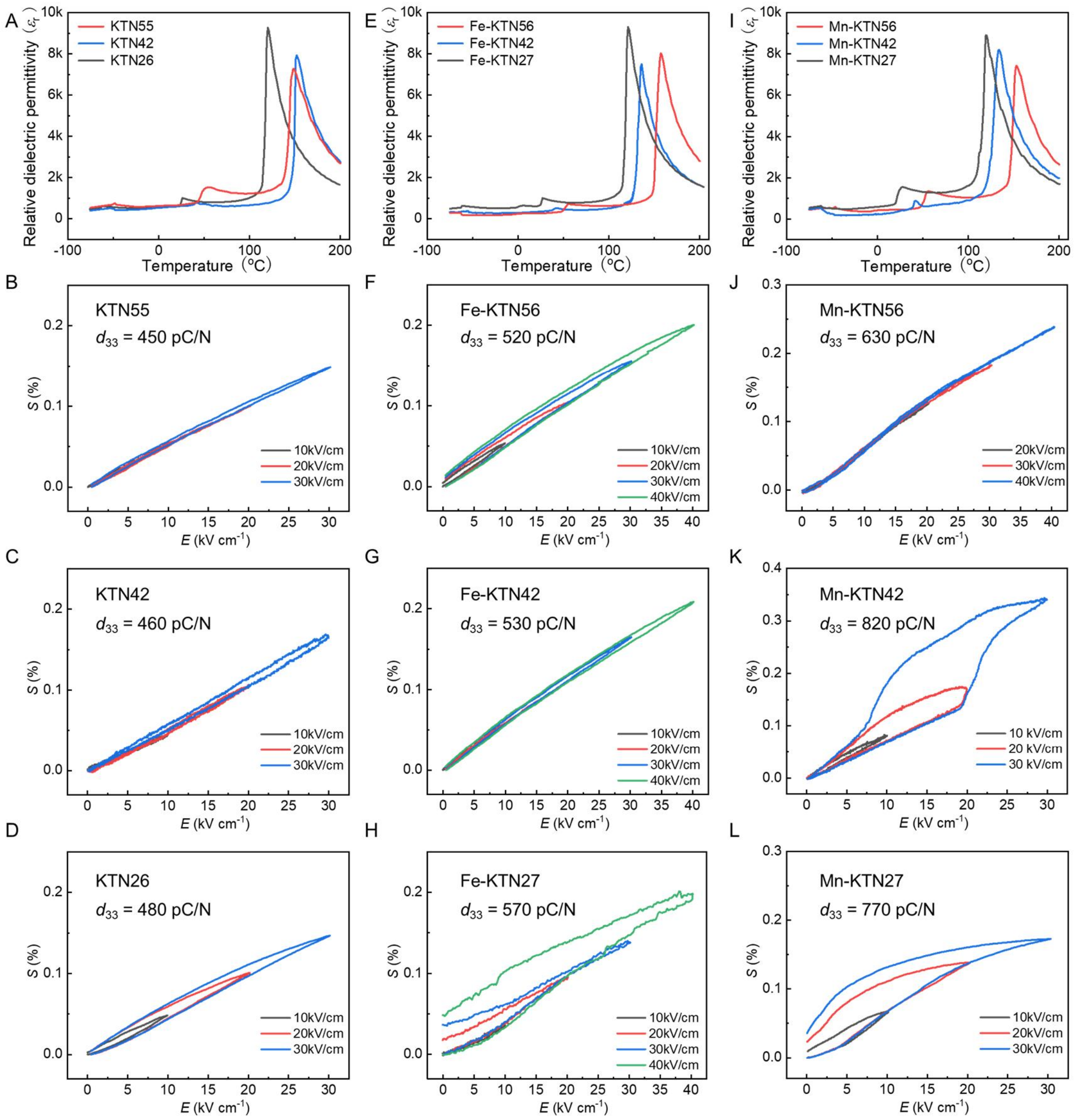

Figure S1. Piezoelectric responses of the pristine, Fe-doped, and Mn-doped KTN samples with different orthorhombic-tetragonal phase transition temperatures $T_{\text{O-T}}$. (A, E, I) Relative dielectric permittivity of the pristine, Fe-doped, and Mn-doped samples. The numbers in the sample names indicate the $T_{\text{O-T}}$ expressed in degrees Celsius. The piezoelectric responses of these samples were measured. (B-D), (F-H), and (J-L) are strain-electric field (S-E) loops of the pristine, Fe-doped, and Mn-doped samples, respectively. The insets are the photos showing the results of the quasi-static d_{33} tests. The legends in (B-D, F-H, and J-L) indicate the maximum applied electric fields.

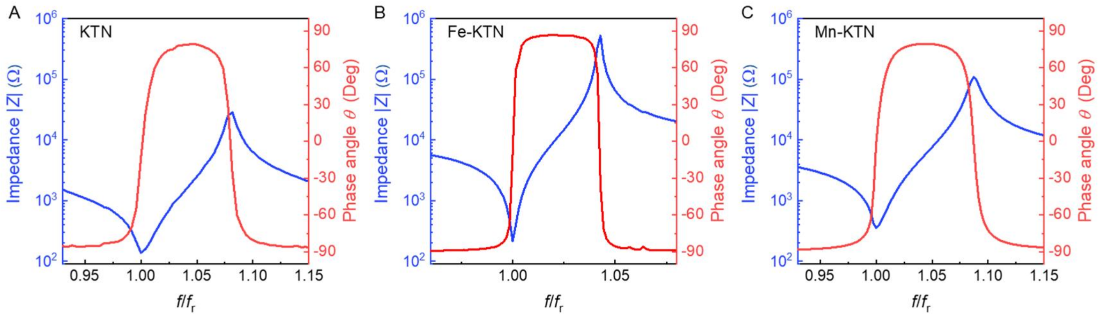

Figure S2. Frequency dependences of impedance (Z) and phase angle (θ) of the (A) pristine, (B) Fe-doped, and (C) Mn-doped KTN samples, characterized through the resonance–antiresonance method using an impedance analyzer at room temperature RT. The mechanical quality factors Q_m were calculated using the equation $Q_m^{-1} = 2\pi f_r RC\left[1-(f_r/f_a)^2\right]$, where f_r is the resonant frequency, f_a is the anti-resonant frequency, R is the resonance resistance, and C is the capacitance at 1 kHz [1]. The Q_m of the pristine, Fe-doped, and Mn-doped KTN samples are 120, 700, and 140, respectively. The same results can also be obtained using the -3 dB method [2].

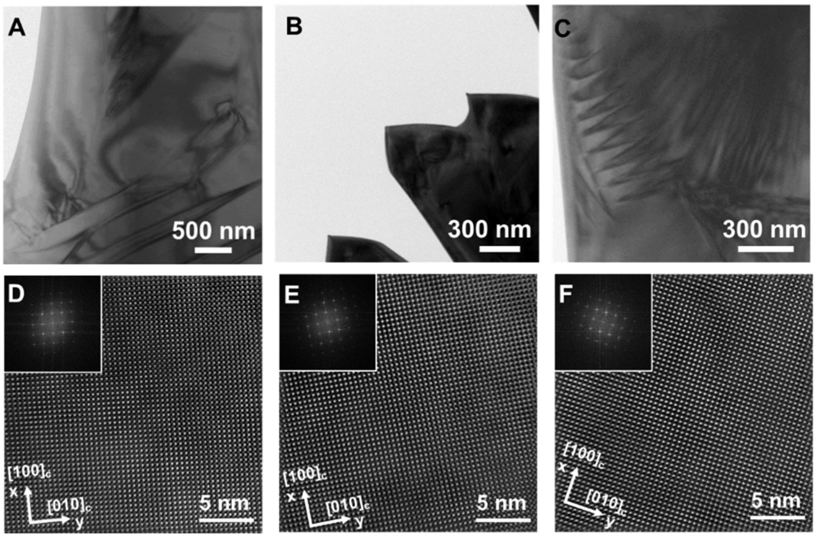

Figure S3. High-resolution transmission electron microscopy (HRTEM) images. (A), (B), and (C) are the bright-field HRTEM images of the irradiated regions of the pristine, Fe-doped, and Mn-doped KTN samples, respectively. (D-F) The corresponding selected area electron diffraction (SAED) patterns of the pristine, Fe-doped, and Mn-doped samples taken along the $[001]_C$ direction, respectively.

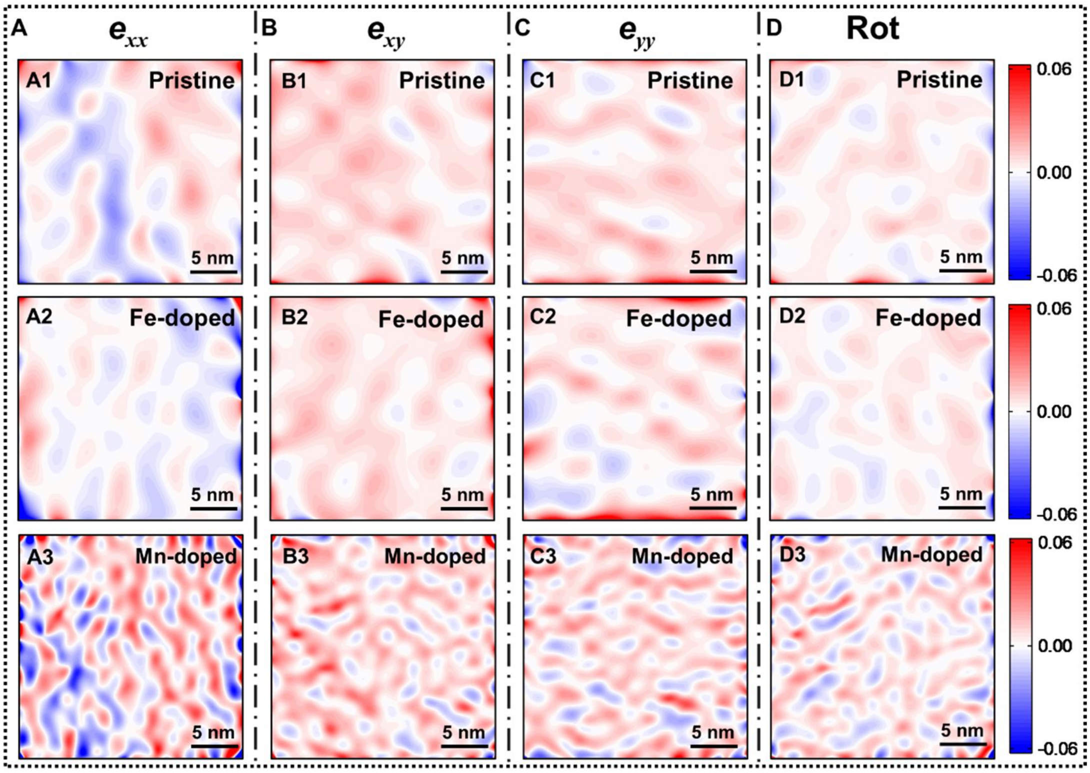

Figure S4. Interface strains estimated using geometric phase analysis (GPA). The strain maps of (A) e_{xx}: in-plane, (B) e_{xy}: shear strain, (C) e_{yy}: out-plane, and (D) Rot: rotation. The serial numbers 1, 2, and 3 represent the pristine, Fe-doped, and Mn-doped KTN samples, respectively.

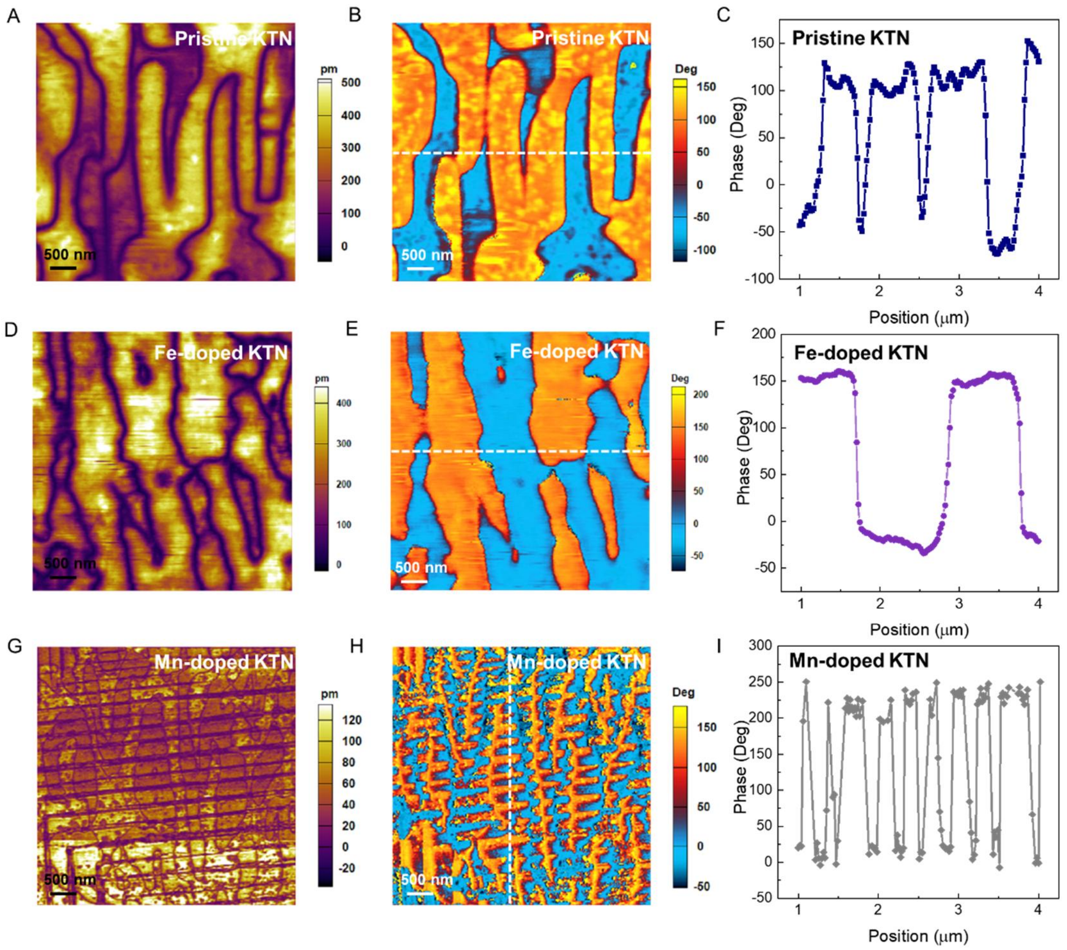

Figure S5. Ferroelectric domain structures observed using piezoresponse force microscopy (PFM). (A, D, G) Amplitude and (B, E, H) phase images of the pristine, Fe-doped, and Mn-doped KTN samples, respectively. (C, F, I) The distributions of phase of the white dashed lines in (B, E, H), showing the characteristic sizes of ferroelectric domains.

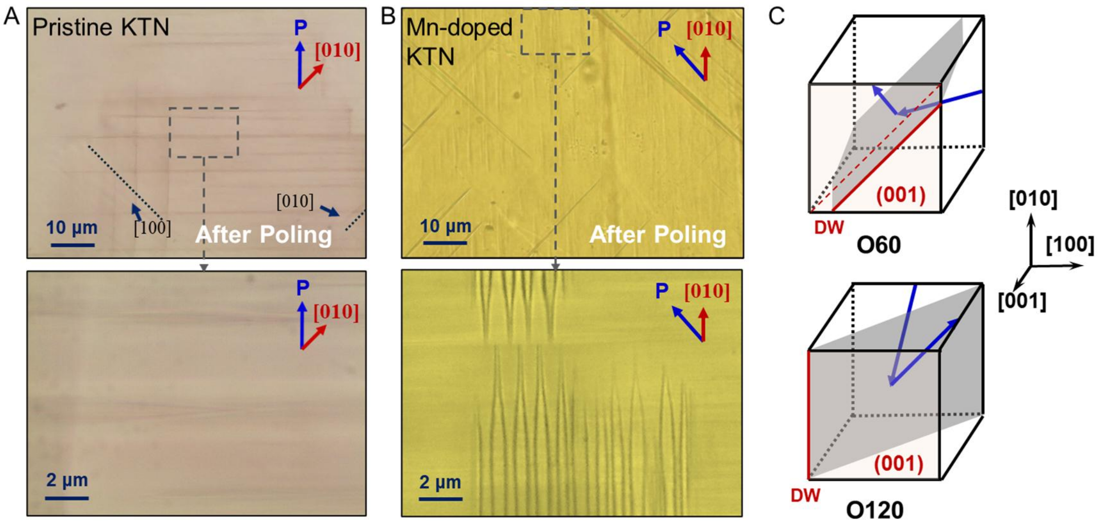

Figure S6. Domain structures observed along the $[001]_C$ direction using polarizing light microscopy. (A, B) are the domain structures in the pristine and Mn-doped KTN samples after poling along the $[001]_C$ direction, respectively. (C) The typical configurations of domain walls after poling along the $[001]_C$ direction. The red solid lines represent the domain walls. In the $[001]_C$-poled samples, the direction of macroscopic polarization remains the same as the $[001]_C$ direction, so that the domain walls are dominated by the O60 and O120. Therefore, the domain walls along the $<110>_C$ and $<010>_C$ directions can be observed along the $[001]_C$ direction. The morphologies of the O60 and O120 domain walls are affected by the total free energy including strain energy, dipole-dipole interaction, and electrostatic energy. The latter two parts are independent of the area of the domain wall. The disordered polarization in the Mn-doped crystal tends to introduce larger dipole-dipole interaction and electrostatic energy, which can be reduced by the curving of the domain walls. Hence, to minimize the total free energy in the Mn-doped crystal, the curved O120 domain walls are increased.

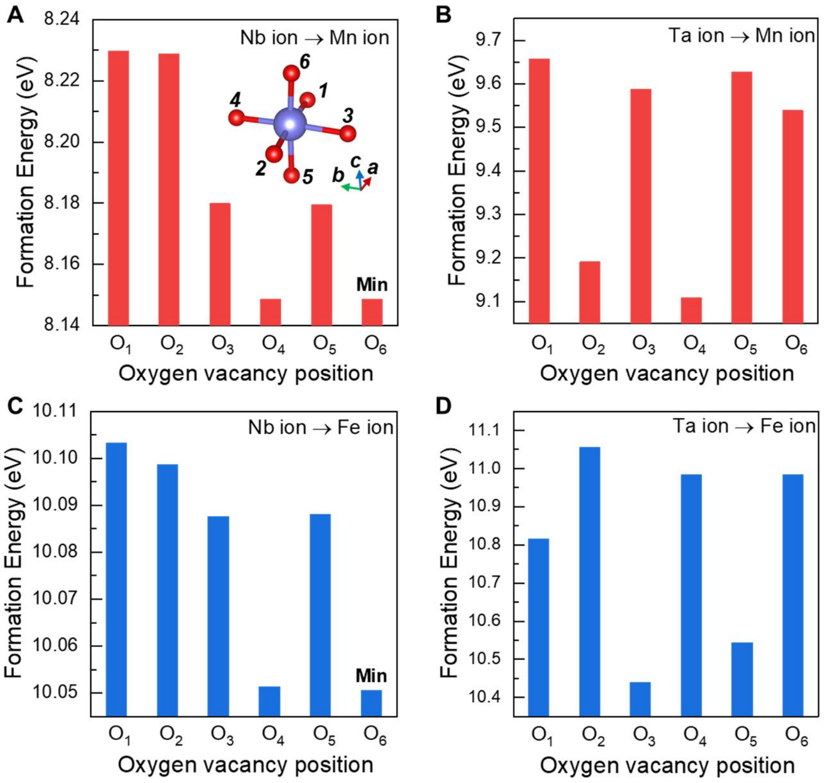

Figure S7 Comparison of formation energies for defect structures in the KTN lattice. (A, B) represent the doping of a Mn ion in sites of Nb and Ta ions, respectively. (C, D) represent the doping of a Fe ion in sites of Nb and Ta ions, respectively. The oxygen sites around the dopant ions are labeled as shown in the illustration in (A). The *a*, *b*, and *c* are the $[100]_C$, $[010]_C$, and $[001]_C$ crystallographic directions, respectively. In a pristine orthorhombic phase KTN lattice, the spontaneous polarization P_s is along the $[011]_C$ direction. The horizontal axis of (A-D) represents the formation of oxygen vacancies $V_O^{\bullet\bullet}$ at the corresponding labeled positions. In terms of the adherence to the principle of the lowest formation energy, the dopant ions tend to displace the Nb ions and the $V_O^{\bullet\bullet}$ tend to form at the O_6 site. The O_6 site is the oxygen site closest to the central B-site ion. This suggests that the defect structure is most stable when the Mn or Fe dopant replaces a Nb ion, coupled with the formation of a $V_O^{\bullet\bullet}$ at the nearest oxygen site. The lowest-energy structures were used in the calculations of electronic configurations.

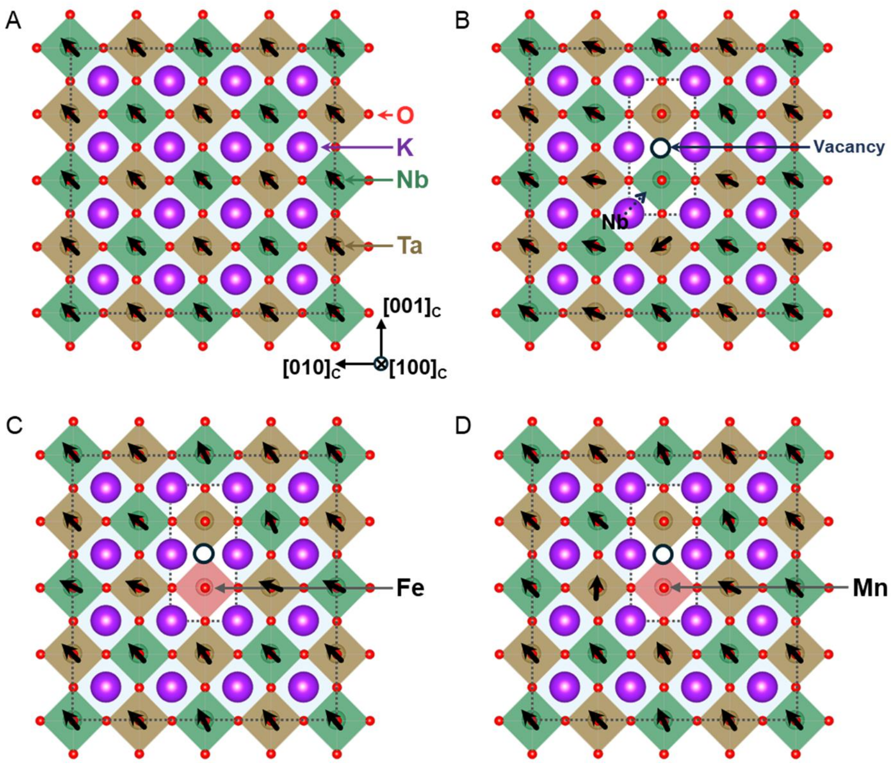

Figure S8. Dipole moment orientations in the octahedra within the $(100)_C$ sections containing the defect dipoles. (A) Pristine KTN lattice, (B) KTN lattice with an oxygen vacancy ($V_O^{\bullet\bullet}$-KTN), (C) Fe-doped lattice, and (D) Mn-doped lattice. The orientations of dipole moments in oxygen octahedra pointing from the negatively charged center to the positively charged center are evaluated by using the simulated structural parameters and marked in the schematics by black arrows. The statistical results of the deviation angle of the dipole moment from the $[011]_C$ direction are presented in Figure 4(E) in the main text.

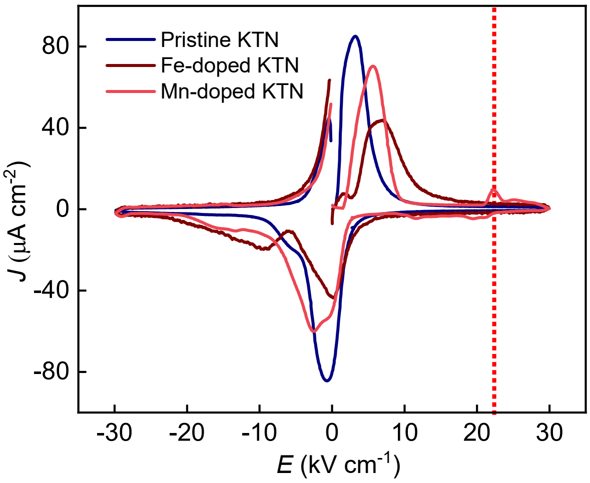

Figure S9. Current and electric field (*J*-*E*) loops under a 1-Hz electric field with a maximum of 30 kV/cm.

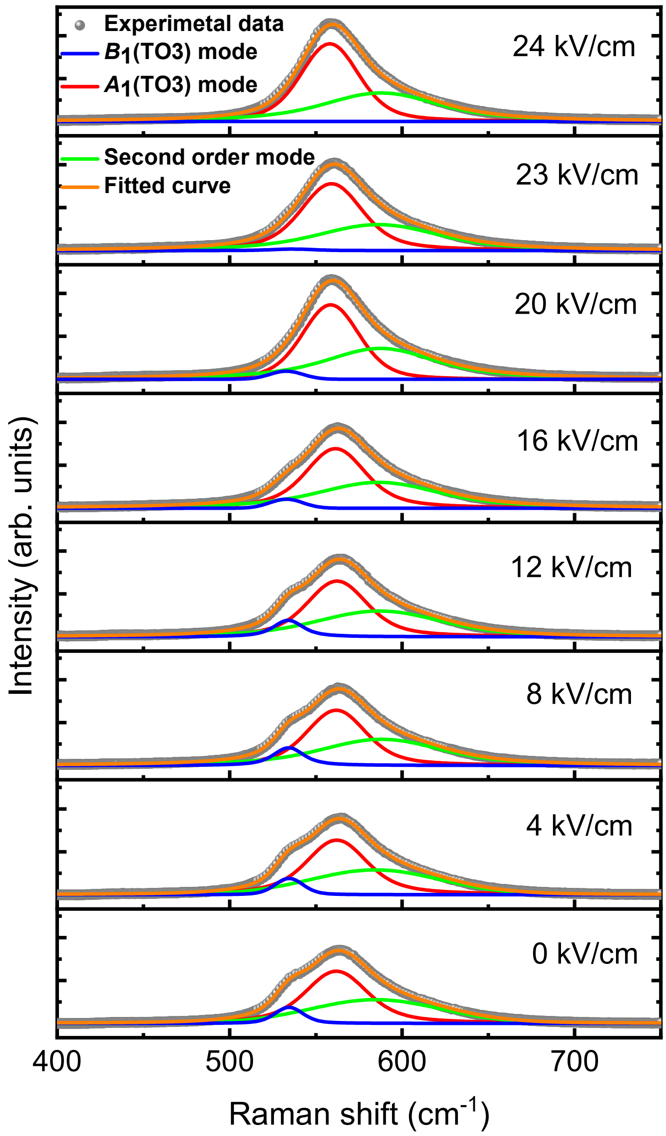

Figure S10. Peak fitting of the Raman spectra of the Mn-doped KTN sample under different electric fields using the Voigt line shape. The blue, red, and green lines represent the B_1(TO3), A_1(TO3), and second-order vibrational modes, respectively. The B_1(TO3) mode almost disappears when the applied electric field reaches 23 kV/cm.

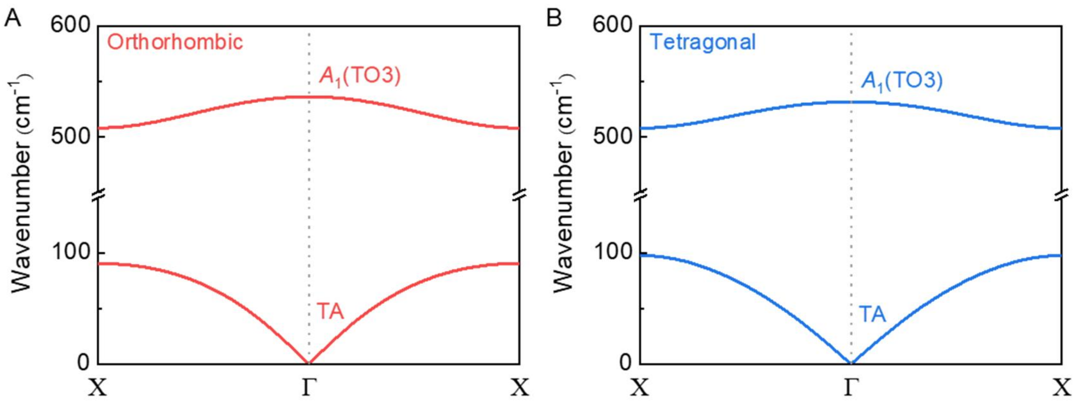

Figure S11. The phonon spectra of the optical A_1(TO3) mode and the acoustic TA mode in (A) orthorhombic KTN and (B) tetragonal KTN, respectively. In the orthorhombic KTN, the wavenumbers of the A_1(TO3) and TA modes at the *X* point of the Brillouin zone are 507 and 90 cm^{-1}, respectively. In the tetragonal KTN, the wavenumbers of the A_1(TO3) and TA modes at the *X* point of the Brillouin zone are 507 and 97 cm^{-1}, respectively. The sum of the two phonons induces the generation of the second-order Raman mode with a Raman shift of ~600 cm^{-1}, which is in good agreement with the ~590 cm^{-1} peak measured in the experiment. This alignment strongly supports the identification of the third fitted peak as a second-order Raman mode.

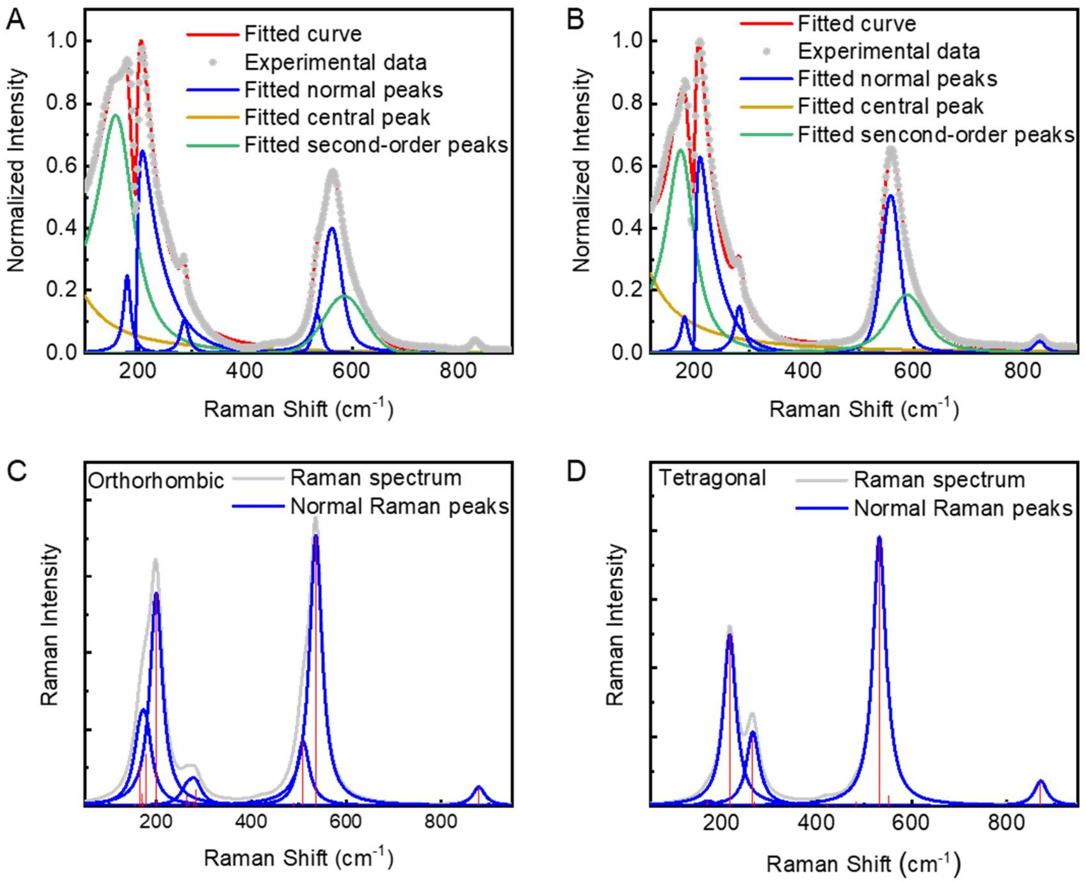

Figure S12. The comparison between experimental and theoretical Raman spectra. (A, B) are the full experimental Raman spectra of orthorhombic and tetragonal KTN crystals, respectively. The normal Raman modes (i.e., the first-order Raman modes), the second-order Raman modes, and the central peak are obtained by fitting. The fitted curves (red lines), containing the contributions from these three parts, are consistent with the experimental results. (C, D) show the calculated Raman spectra of orthorhombic and tetragonal KTN crystals, respectively. The red sticks indicate the calculated Raman intensities of the normal modes (i.e., the first-order modes). The blue lines are computed by applying a Gaussian broadening of 80 cm^{-1} to the discrete spectrum. The grey line is the sum of the blue lines.

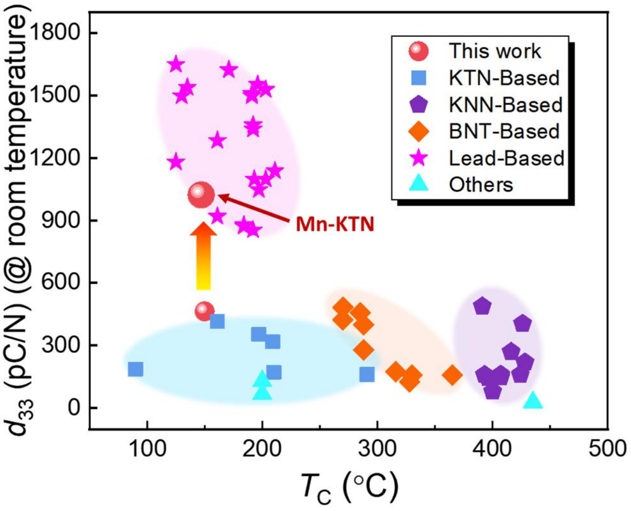

Figure S13. The d_{33} of the partial mainstream piezoelectric crystals. The red dots represent the results in the work, and the arrow shows the enhancement in the piezoelectric response after Mn doping. The data of KTN-based, KNN-based, BNT-based, lead-based, and other materials, cited in the figure, were from references [3-7], [8-14], [15-22], [23-29], and [30,31], respectively.

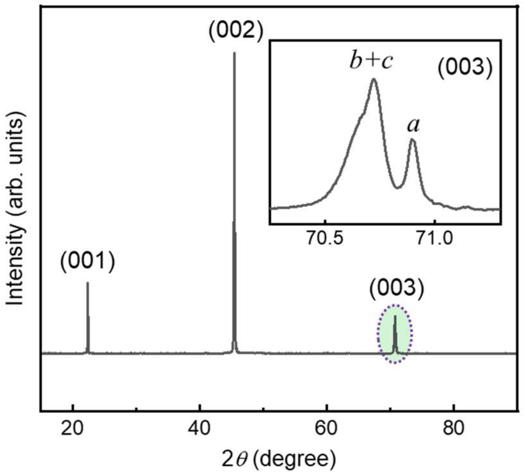

Figure S14. X-ray (λ = 1.5406 Å) diffraction pattern collected at the $(001)_C$ facet at room temperature (X'Pert PRO MPD, PANalytical). The inset shows the splitting of the $(003)_C$ peak. Lattice constants of a = 3.985 Å and $b \approx c$ = 3.992 Å are obtained.

Table S1. Compositional analysis of the pristine, Fe-doped, and Mn-doped KTN samples, characterized by an electron probe X-ray microanalyzer. Here, the content of a certain ion is its proportion to the total number of B-site ions. The compositions are described using the way of $\mu \pm \sigma$, where μ is the average value and σ is the sample standard deviation.

Samples	Nb content (mol%)	Ta content (mol%)	Dopant content (mol%)
Pristine KTN	58.04 ± 0.79	41.96 ± 0.79	-
Fe-doped KTN	57.23 ± 0.86	42.23 ± 0.86	0.54 ± 0.08
Mn-doped KTN	57.33 ± 0.84	42.13 ± 0.84	0.53 ± 0.09

Table S2. The ionic radius of K, Nb, Ta, Mn, Fe, and O ions [32]

Ion species	Coordination Number	Ionic Radius (Å)
K^{+}	12	1.64
Nb^{5+}	6	0.64
Ta^{5+}	6	0.64
Mn^{2+}	6	0.83
Mn^{3+}	6	0.645
Mn^{4+}	6	0.53
Fe^{2+}	6	0.78
Fe^{3+}	6	0.645
O^{2-}	6	1.40

Table S3. Goldschmidt tolerance factor t and the stability of corresponding perovskite structures. The Goldschmidt tolerance factor t, equal to $(R_A + R_X)/\sqrt{2}(R_B + R_X)$, is an approved criterion for predicting the stability of the perovskite structure based on the chemical formula, ABX_3, and the ionic radii, R_i, of each ion (A, B, X). The ionic radius R_i (A, B, X) of KTN-based materials is presented in Table S2. Empirically, the t is between 0.81 and 1.11 for the stable perovskite structure[33]. The evaluated t indicates that the structures of the Fe and Mn dopants occupying the B-sites are stable from an empirical point of view.

Ion species	Position	t	Stability
Nb^{5+}/ Ta^{5+}	B-site	1.053	Stable
Mn^{2+}	B-site	0.964	Stable
Mn^{3+}	B-site	1.051	Stable
Mn^{4+}	B-site	1.114	Stable
Fe^{2+}	B-site	0.986	Stable
Fe^{3+}	B-site	1.051	Stable

Table S4. Total magnetic moments μ_{tot} (μ_B) of the simulated structures.

System	μ_{tot} (μ_B)
Pristine KTN	0
KTN with the oxygen vacancy	1.634
Fe-doped KTN	2.873
Mn-doped KTN	3.798

Table S5. Magnetic moment μ (μ_B) of each atom of pristine KTN with the oxygen vacancy. Here, the atoms are listed in the same order as the atoms in the POSCAR file shown in Appendix S2. In contrast, the pristine KTN crystal is non-magnetic, with each atom having a magnetic moment of zero.

atom	$\mu(\mu_B)$	atom	$\mu(\mu_B)$	atom	$\mu(\mu_B)$	atom	$\mu(\mu_B)$	atom	$\mu(\mu_B)$	atom	$\mu(\mu_B)$	atom	$\mu(\mu_B)$	atom	$\mu(\mu_B)$
K	0.001	K	0.001	Nb	0.054	O	-0.004	O	-0.002	O	-0.005	O	-0.01	O	-0.001
K	0	K	0.001	Nb	0.055	O	-0.003	O	-0.001	O	-0.005	O	-0.008	O	-0.001
K	0.001	K	0	Nb	0.052	O	-0.008	O	-0.001	O	-0.002	O	-0.001	O	-0.002
K	0.001	K	0	Nb	0.016	O	-0.002	O	-0.004	O	-0.002	O	-0.002	O	-0.002
K	0.001	K	0.001	Nb	0.053	O	-0.008	O	-0.001	O	-0.002	O	-0.005	O	-0.01
K	0.001	K	0	Nb	0.017	O	-0.008	O	-0.004	O	-0.001	O	-0.005	O	-0.002
K	0.001	K	0.001	Nb	0.019	O	-0.008	O	-0.004	O	-0.002	O	-0.002	O	-0.002
K	0	K	0.001	Nb	0.022	O	-0.001	O	-0.003	O	-0.007	O	-0.005	Ta	0.01
K	0	K	0	Nb	0.051	O	-0.002	O	-0.002	O	-0.002	O	-0.002	Ta	0.009
K	0.001	K	0.001	Nb	0.111	O	-0.002	O	-0.001	O	-0.011	O	-0.002	Ta	0.011
K	0.001	K	0.001	Nb	0.021	O	-0.002	O	-0.002	O	-0.002	O	-0.002	Ta	0.019
K	0.001	K	0.001	Nb	0.094	O	-0.001	O	-0.005	O	-0.002	O	-0.002	Ta	0.065
K	0	K	0.001	Nb	0.017	O	-0.002	O	-0.001	O	-0.002	O	-0.005	Ta	0.045
K	0.001	K	0.001	Nb	0.102	O	-0.001	O	-0.001	O	-0.002	O	-0.009	Ta	0.027
K	0	K	0.001	Nb	0.016	O	-0.002	O	-0.001	O	-0.006	O	-0.002	Ta	0.057
K	0.001	K	0	Nb	0.02	O	-0.002	O	-0.001	O	-0.011	O	-0.008	Ta	0.009
K	0	K	0.001	O	-0.002	O	-0.003	O	-0.001	O	-0.002	O	-0.002	Ta	0.008
K	0	K	0.001	O	-0.001	O	-0.002	O	-0.001	O	-0.005	O	-0.01	Ta	0.028
K	0	K	0.001	O	-0.002	O	-0.008	O	-0.001	O	-0.005	O	-0.001	Ta	0.01
K	0.001	K	0.001	O	-0.001	O	-0.004	O	-0.004	O	-0.002	O	-0.01	Ta	0.008
K	0	K	0.001	O	-0.002	O	-0.001	O	-0.001	O	-0.011	O	-0.001	Ta	0.01
K	0.001	K	0	O	-0.001	O	-0.001	O	-0.002	O	-0.007	O	-0.002	Ta	0.028
K	0.001	K	0.001	O	-0.004	O	-0.005	O	-0.001	O	-0.002	O	-0.002	Ta	0.042
K	0	K	0.001	O	-0.005	O	-0.006	O	-0.003	O	-0.002	O	-0.001	Ta	0.01
K	0	Nb	0.021	O	-0.004	O	-0.002	O	-0.004	O	-0.002	O	-0.002	Ta	0.027
K	0.001	Nb	0.019	O	-0.003	O	-0.009	O	-0.002	O	-0.002	O	-0.005	Ta	0.008
K	0	Nb	0.069	O	-0.004	O	-0.001	O	-0.001	O	-0.005	O	-0.005	Ta	0.009
K	0.001	Nb	0.112	O	-0.001	O	-0.001	O	-0.001	O	-0.002	O	-0.002	Ta	0.028
K	0	Nb	0.021	O	-0.001	O	-0.001	O	-0.002	O	-0.005	O	-0.002	Ta	0.027
K	0	Nb	0.111	O	-0.001	O	-0.009	O	-0.004	O	-0.002	O	-0.001	Ta	0.026
K	0.001	Nb	0.019	O	-0.001	O	-0.002	O	-0.001	O	-0.005	O	-0.001	Ta	0.009
K	0.001	Nb	0.018	O	-0.001	O	-0.001	O	-0.002	O	-0.011	O	-0.002	Ta	0.011
K	0.001	Nb	0.056	O	-0.001	O	-0.002	O	-0.007	O	-0.002	O	-0.009	Ta	0.053
K	0	Nb	0.017	O	-0.001	O	-0.002	O	-0.01	O	-0.002	O	-0.001	Ta	0.011
K	0.001	Nb	0.051	O	-0.002	O	-0.004	O	-0.002	O	-0.005	O	-0.002	Ta	0.029
K	0	Nb	0.015	O	-0.001	O	-0.004	O	-0.005	O	-0.002	O	-0.002	Ta	0.042
K	0.001	Nb	0.017	O	-0.001	O	-0.004	O	-0.002	O	-0.005	O	-0.001	Ta	0.011

K	0	Nb	0.069	O	-0.002	O	-0.002	O	-0.005	O	-0.005	O	-0.002	Ta	0.009
K	0.001	Nb	0.048	O	-0.001	O	-0.001	O	-0.002	O	-0.001	O	-0.01	Ta	0.052
K	0	Nb	0.055	O	-0.001	O	-0.001	O	-0.001	O	-0.002	O	-0.001	Total	1.634

Table S6. Magnetic moment μ (μ_B) of each atom of Fe-doped KTN. Here, the atoms are listed in the same order as the atoms in the POSCAR file shown in Appendix S3.

atom	$\mu(\mu_B)$	atom	$\mu(\mu_B)$	atom	$\mu(\mu_B)$	atom	$\mu(\mu_B)$	atom	$\mu(\mu_B)$	atom	$\mu(\mu_B)$	atom	$\mu(\mu_B)$	atom	$\mu(\mu_B)$
Fe	2.711	K	0	Nb	0.003	O	0	O	0	O	0	O	0	O	0.001
K	0	K	0	Nb	0.001	O	0	O	0	O	0	O	-0.001	O	0
K	0	K	0	Nb	0	O	0	O	0	O	0	O	0	O	0
K	0	K	0	Nb	0	O	0	O	0	O	0	O	0	O	0
K	0	K	0	Nb	0	O	0	O	0	O	0	O	0	O	0.002
K	0.001	K	0	Nb	0.001	O	0.001	O	0	O	0	O	0	O	0
K	0	K	0	Nb	0	O	0	O	0	O	0	O	0	O	0
K	0	K	0	Nb	0	O	0	O	0	O	0	O	0	Ta	0
K	0	K	0	Nb	0	O	0	O	0	O	0	O	0	Ta	0
K	0	K	0	Nb	0	O	0	O	0	O	0	O	0	Ta	0
K	0	K	0	Nb	0.001	O	0	O	0	O	0	O	0	Ta	0
K	0	K	0	Nb	-0.002	O	0	O	0.003	O	0	O	0	Ta	0.016
K	0	K	0	Nb	0.001	O	0	O	0.002	O	0	O	0	Ta	0
K	0	K	0	Nb	0	O	0	O	0	O	0.001	O	0	Ta	0
K	0	K	0	Nb	0.001	O	0	O	0	O	0	O	0	Ta	0
K	0	K	0	Nb	0	O	0	O	0	O	0	O	-0.002	Ta	0
K	0	K	0	O	0	O	0	O	0	O	0	O	0	Ta	0
K	0	K	0	O	-0.001	O	0	O	0	O	0	O	0	Ta	0
K	0	K	0	O	0	O	0.001	O	0	O	0	O	0	Ta	0
K	0	K	-0.001	O	0	O	0	O	0	O	0	O	0	Ta	0
K	0	K	0	O	0	O	0	O	-0.001	O	0	O	0	Ta	0
K	0	K	0	O	0	O	0	O	0.095	O	0	O	0	Ta	0
K	0	K	0	O	0	O	0	O	0	O	0	O	0	Ta	-0.006
K	0	K	0	O	0	O	0.074	O	0	O	0	O	0	Ta	0
K	0	K	0	O	0	O	0	O	0	O	0	O	0	Ta	0
K	0	Nb	0	O	0	O	0	O	0	O	0	O	-0.021	Ta	0
K	0	Nb	0	O	0	O	0	O	0	O	0	O	-0.018	Ta	0.002
K	0	Nb	-0.001	O	0	O	0.001	O	0	O	0	O	0	Ta	0
K	0	Nb	0	O	0	O	0	O	0	O	0	O	0	Ta	0
K	0	Nb	-0.001	O	0	O	0	O	0	O	0	O	0	Ta	0
K	0	Nb	0	O	0	O	0	O	0	O	0	O	0	Ta	0
K	0	Nb	0.002	O	0.008	O	0	O	0	O	0	O	0	Ta	0
K	0	Nb	0	O	0	O	0	O	0.001	O	0	O	0	Ta	0
K	0	Nb	0	O	0.001	O	0	O	0	O	0	O	0	Ta	0
K	0	Nb	0	O	0	O	0	O	0	O	0	O	0	Ta	0.006
K	0	Nb	-0.002	O	0	O	0	O	0	O	0	O	0	Ta	-0.006

K	0	Nb	0	O	0	O	0	O	0	O	0	O	0.001	Ta	0
K	0	Nb	0	O	0	O	0.001	O	0.001	O	0	O	0	Ta	0
K	0	Nb	-0.002	O	0	O	0	O	0.002	O	0.001	O	0	Ta	0
K	-0.001	Nb	0	O	0	O	0	O	0	O	0.001	O	0	Total	2.873

Table S7. Magnetic moment μ (μ_B) of each atom of Mn-doped KTN. Here, the atoms are listed in the same order as the atoms in the POSCAR file shown in Appendix S4.

atom	$\mu(\mu_B)$	atom	$\mu(\mu_B)$	atom	$\mu(\mu_B)$	atom	$\mu(\mu_B)$	atom	$\mu(\mu_B)$	atom	$\mu(\mu_B)$	atom	$\mu(\mu_B)$	atom	$\mu(\mu_B)$
Mn	3.667	K	0	Nb	0	O	0	O	0	O	0	O	0	O	0
K	0	K	0	Nb	0.002	O	0	O	0	O	0.001	O	0	O	0
K	0	K	0	Nb	0	O	0	O	0	O	0	O	0	O	0
K	0	K	0	Nb	0	O	0	O	0	O	0	O	0	O	0
K	0	K	0	Nb	0	O	0	O	0	O	0	O	0	O	0.001
K	0	K	0	Nb	0.001	O	0	O	0	O	0	O	0	O	0
K	0	K	0	Nb	0	O	0	O	0	O	0.001	O	0	O	0
K	0	K	0	Nb	0	O	0	O	0	O	0	O	0	Ta	0
K	0	K	0	Nb	0.002	O	0	O	0	O	0	O	0	Ta	0
K	0	K	0	Nb	0	O	0	O	0	O	0	O	0	Ta	0
K	0	K	0	Nb	0.002	O	0	O	0	O	0	O	0	Ta	0
K	0	K	-0.001	Nb	0	O	0	O	0	O	0	O	0	Ta	0
K	0	K	0	Nb	-0.001	O	0	O	0	O	0	O	0	Ta	0
K	0	K	0	Nb	0.001	O	0	O	0.003	O	0	O	0	Ta	0
K	0	K	0	Nb	-0.001	O	0	O	0.003	O	0	O	0	Ta	0
K	0	K	0	Nb	0	O	0	O	0	O	0	O	0	Ta	0.026
K	0	K	0	O	0	O	0	O	0	O	0	O	0	Ta	0
K	0	K	0	O	-0.007	O	0	O	0	O	0	O	0	Ta	0
K	0	K	0	O	-0.002	O	0	O	0	O	0	O	-0.002	Ta	0
K	0	K	0	O	0	O	0	O	0	O	0	O	0	Ta	0
K	0	K	-0.001	O	0	O	0	O	0	O	0	O	0	Ta	0
K	0	K	0	O	0	O	0	O	0	O	0	O	0	Ta	0
K	0	K	0	O	0	O	0	O	0	O	0	O	0	Ta	0
K	0	K	0	O	0	O	0	O	-0.001	O	0	O	0	Ta	0
K	0	K	0	O	0	O	0.067	O	0.002	O	0	O	-0.004	Ta	0.005
K	0	Nb	0.002	O	0	O	0	O	0	O	0	O	-0.004	Ta	0.005
K	0	Nb	0	O	0	O	0	O	0	O	0	O	0	Ta	0
K	0	Nb	0.002	O	0	O	0	O	0	O	0	O	0	Ta	0
K	0	Nb	0.002	O	0	O	0	O	0	O	0	O	-0.002	Ta	0.005
K	0	Nb	0	O	0	O	0	O	0	O	0	O	0	Ta	0
K	0	Nb	0	O	0	O	0.001	O	0	O	0	O	0	Ta	0
K	0	Nb	0	O	0	O	0	O	0	O	0	O	0	Ta	0
K	0	Nb	0	O	0	O	0	O	0.003	O	0	O	0.001	Ta	0
K	0	Nb	0	O	0	O	0	O	0	O	0	O	0	Ta	0
K	0	Nb	0	O	0	O	0	O	0.003	O	0	O	0	Ta	0

K	0	Nb	0.002	O	0	O	0	O	0	O	0	O	0	Ta	0.004
K	0	Nb	0	O	0	O	0	O	0	O	0	O	0	Ta	0
K	0	Nb	0	O	0	O	0	O	0	O	0	O	0	Ta	0
K	0	Nb	0	O	0	O	0	O	0	O	0.001	O	0.002	Ta	0
K	0	Nb	0	O	0	O	0	O	0	O	0.001	O	0	Total	3.798

References

1 Yang, Y. et al. Defect engineering in barium titanate ferroelectric ceramic showing simultaneous enhancement of piezoelectric coefficient and mechanical quality factor. *J. Eur. Ceram. Soc.* **44**, 891-897 (2024).

2 Zhang, S., Lebrun, L., Randall, C. A. & Shrout, T. R. Orientation dependence properties of modified tetragonal $0.88Pb(Zn_{1/3}Nb_{2/3})O_3$–$0.12PbTiO_3$ single crystals. *Phys. Status Solidi A* **202**, 151-157 (2004).

3 Huo, X. et al. A high quality lead-free (Li, Ta) modified (K, Na)NbO_3 single crystal and its complete set of elastic, dielectric and piezoelectric coefficients with macroscopic 4mm symmetry. *CrystEngComm* **16**, 9828-9833 (2014).

4 Zheng, L. et al. Large size lead-free (Na,K)(Nb,Ta)O_3 piezoelectric single crystal: growth and full tensor properties. *CrystEngComm* **15**, 7718-7722 (2013).

5 Cao, X. et al. Defect dipole evolution and its impact on the ferroelectric properties of Fe-doped KTN single crystals. *J. Am. Ceram. Soc.* **102**, 3117-3122 (2019).

6 Tian, H. et al. Origin of giant piezoelectric effect in lead-free $K_{1-x}Na_xTa_{1-y}Nb_yO_3$ single crystals. *Sci. Rep.-UK* **6**, 25637 (2016).

7 Tian, H., Hu, C., Meng, X., Zhou, Z. & Shi, G. Dielectric, piezoelectric, and elastic properties of $K_{0.8}Na_{0.2}NbO_3$ single crystals. *J. Mater. Chem. C* **3**, 9609-9614 (2015).

8 Chen, K., Xu, G., Yang, D., Wang, X. & Li, J. Dielectric and piezoelectric properties of lead-free $0.95(K_{0.5}Na_{0.5})NbO_3$–$0.05LiNbO_3$ crystals grown by the Bridgman method. *J. Appl. Phys.* **101**, 044103 (2007).

9 Lin, D., Li, Z., Zhang, S., Xu, Z. & Yao, X. Dielectric/piezoelectric properties and temperature dependence of domain structure evolution in lead free single crystal. *Solid State Commun.* **149**, 1646-1649 (2009).

10 Song, J. et al. Enhanced piezoelectric property and microstructure of large $CaZrO_3$-doped $Na_{0.5}K_{0.5}NbO_3$-based single crystal with 20 mm over. *Mater. Lett.*

204, 19-22 (2017).

11 Li, X. et al. The Growth and Properties of Lead-Free Ferroelectric Single Crystals. *Crystals* **5**, 172-192 (2015).

12 Hu, C. et al. High-quality $K_{0.47}Na_{0.53}NbO_3$ single crystal toward high performance transducer. *RSC Adv.* **7**, 7003-7007 (2017).

13 Inagaki, Y. & Kakimoto, K. Dielectric and Piezoelectric Properties of Mn-Doped $Na_{0.5}K_{0.5}NbO_3$ Single Crystals Grown by Flux Method. *Appl. Phys. Express* **1**, 061602 (2008).

14 Huo, X. et al. (K, Na, Li)(Nb, Ta)O_3: Mn Lead-Free Single Crystal with High Piezoelectric Properties. *J. Am. Ceram. Soc.* **98**, 1829-1835 (2015).

15 Sun, R. et al. Growth and orientation dependence of electrical properties of $0.92Na_{0.5}Bi_{0.5}TiO_3$-$0.08K_{0.5}Bi_{0.5}TiO_3$ lead-free piezoelectric single crystal. *J. Appl. Phys.* **109**, 124113 (2011).

16 Ge, W. et al. Crystal growth and high piezoelectric performance of $0.95Na_{0.5}Bi_{0.5}TiO_3$–$0.05BaTiO_3$ lead-free ferroelectric materials. *J. Phys. D Appl. Phys.* **41**, 115403 (2008).

17 Ge, W. et al. Evolution of structure in $Na_{0.5}Bi_{0.5}TiO_3$ single crystals with $BaTiO_3$. *Appl. Phys. Lett.* **105**, 162913 (2014).

18 Zhang, Q., Zhao, X., Sun, R. & Luo, H. Crystal growth and electric properties of lead-free NBT-BT at compositions near the morphotropic phase boundary. *Phys. Status Solidi A* **208**, 1012-1020 (2011).

19 Xiao, J. et al. Temperature-induced phase transition of <001>-oriented $0.92Na_{0.5}Bi_{0.5}TiO_3$-$0.08Bi(Zn_{0.5}Ti_{0.5})O_3$ lead-free single crystals. *Ceram. Int.* **47**, 14062-14066 (2021).

20 Zhang, Q. et al. Enhanced piezoelectric and ferroelectric properties in Mn-doped $Na_{0.5}Bi_{0.5}TiO_3$–$BaTiO_3$ single crystals. *Appl. Phys. Lett.* **95**, 102904 (2009).

21 Sun, R. et al. Dielectric, electromechanical coupling properties of Mn-doped $Na_{0.5}Bi_{0.5}TiO_3$–$BaTiO_3$ lead-free single crystal. *Appl. Phys. A* **103**, 199-205 (2010).

22 Yi, X. et al. Flux growth and characterization of lead-free piezoelectric single crystal $[Bi_{0.5}(Na_{1-x}K_x)_{0.5}]TiO_3$. *J. Cryst. Growth* **281**, 364-369 (2005).

23 Yang, S. et al. Textured ferroelectric ceramics with high electromechanical coupling factors over a broad temperature range. *Nat. Commun.* **12**, 1414 (2021).

24 Li, Q. et al. Enhanced Piezoelectric Properties and Improved Property Uniformity in Nd-Doped PMN-PT Relaxor Ferroelectric Single Crystals. *Adv. Funct. Mater.* **32**, 2201719 (2022).

25 Zhang, S., Lee, S.-M., Kim, D.-H., Lee, H.-Y. & Shrout, T. R. Elastic, Piezoelectric, and Dielectric Properties of $0.71Pb(Mg_{1/3}Nb_{2/3})O_3$–$0.29PbTiO_3$ Crystals Obtained by Solid-State Crystal Growth. *J. Am. Ceram. Soc.* **91**, 683-686 (2008).

26 Zhang, S. et al. Advantages and challenges of relaxor-$PbTiO_3$ ferroelectric crystals for electroacoustic transducers – A review. *Prog. Mater. Sci.* **68**, 1-66 (2015).

27 Sun, E., Zhang, R., Wu, F., Yang, B. & Cao, W. Influence of manganese doping to the full tensor properties of $0.24Pb(In_{1/2}Nb_{1/2})O_3$-$0.47Pb(Mg_{1/3}Nb_{2/3})O_3$-$0.29PbTiO_3$ single crystals. *J. Appl. Phys.* **113**, 074108 (2013).

28 Liu, J. et al. Impact of alternating current electric field poling on piezoelectric and dielectric properties of $Pb(In_{1/2}Nb_{1/2})O_3$–$Pb(Mg_{1/3}Nb_{2/3})O_3$–$PbTiO_3$ ferroelectric crystals. *J. Appl. Phys.* **128**, 094104 (2020).

29 Zhang, S., Lee, S.-M., Kim, D.-H., Lee, H.-Y. & Shrout, T. R. Characterization of Mn-modified $Pb(Mg_{1/3}Nb_{2/3})O_3$–$PbZrO_3$–$PbTiO_3$ single crystals for high power broad bandwidth transducers. *Appl. Phys. Lett.* **93**, 122908 (2008).

30 Zgonik, M. et al. Materials constants of $KNbO_3$ relevant for electro- and acousto-optics. *J. Appl. Phys.* **74**, 1287-1297 (1993).

31 Jiang, W., Cao, W., Yi, X. & Chen, H. The elastic and piezoelectric properties of tungsten bronze ferroelectric crystals $(Sr_{0.7}Ba_{0.3})_2NaNb_5O_{15}$ and $(Sr_{0.3}Ba_{0.7})_2NaNb_5O_{15}$. *J. Appl. Phys.* **97**, 094106 (2005).

32 Shannon, R. D. Revised effective ionic radii and systematic studies of interatomic distances in halides and chalcogenides. *Acta Cryst.* **32**, 751-767 (1976).

33 Green, M. A., Ho-Baillie, A. & Snaith, H. J. The emergence of perovskite solar cells. *Nat. Photonics* **8**, 506-514 (2014).

Appendix S1. POSCAR file of the 4×4×4 supercell of pristine KTN

==

```
KTN
1.0
       15.9399995804         0.0000000000         0.0000000000
        0.0000000000        15.9695816040         0.0000000000
        0.0000000000         0.0000000000        15.9695816040
    K    Nb     O    Ta
   64    32   192    32
Direct
     0.374976000         0.373805000         0.126195000
     0.374976000         0.623805000         0.376195000
     0.374976000         0.623805000         0.876195000
     0.874976000         0.123805000         0.376195000
     0.874976000         0.123805000         0.876195000
     0.874976000         0.623805000         0.376195000
     0.125024000         0.123805000         0.376195000
     0.125024000         0.123805000         0.876195000
     0.125024000         0.623805000         0.376195000
     0.125024000         0.623805000         0.876195000
     0.625024000         0.123805000         0.376195000
     0.625024000         0.123805000         0.876195000
     0.625024000         0.623805000         0.376195000
     0.625024000         0.623805000         0.876195000
     0.124976000         0.123805000         0.126195000
     0.124976000         0.123805000         0.626195000
     0.124976000         0.623805000         0.126195000
     0.124976000         0.623805000         0.626195000
     0.624976000         0.123805000         0.126195000
     0.624976000         0.123805000         0.626195000
     0.624976000         0.623805000         0.126195000
     0.624976000         0.623805000         0.626195000
     0.375024000         0.123805000         0.126195000
     0.375024000         0.123805000         0.626195000
     0.375024000         0.623805000         0.126195000
     0.375024000         0.623805000         0.626195000
     0.875024000         0.123805000         0.126195000
     0.875024000         0.123805000         0.626195000
     0.875024000         0.623805000         0.126195000
     0.875024000         0.623805000         0.626195000
     0.374976000         0.123805000         0.876195000
     0.374976000         0.123805000         0.376195000
     0.874976000         0.623805000         0.876195000
     0.875024000         0.873805000         0.376195000
     0.374976000         0.373805000         0.626195000
     0.374976000         0.873805000         0.126195000
     0.374976000         0.873805000         0.626195000
     0.874976000         0.373805000         0.126195000
     0.874976000         0.373805000         0.626195000
     0.874976000         0.873805000         0.126195000
     0.874976000         0.873805000         0.626195000
     0.125024000         0.373805000         0.126195000
     0.875024000         0.873805000         0.876195000
     0.125024000         0.873805000         0.126195000
     0.125024000         0.873805000         0.626195000
```

0.625024000	0.373805000	0.126195000
0.625024000	0.373805000	0.626195000
0.625024000	0.873805000	0.126195000
0.625024000	0.873805000	0.626195000
0.125024000	0.373805000	0.626195000
0.124976000	0.373805000	0.876195000
0.124976000	0.373805000	0.376195000
0.875024000	0.373805000	0.876195000
0.875024000	0.373805000	0.376195000
0.375024000	0.873805000	0.876195000
0.375024000	0.873805000	0.376195000
0.375024000	0.373805000	0.376195000
0.375024000	0.373805000	0.876195000
0.624976000	0.873805000	0.376195000
0.624976000	0.373805000	0.876195000
0.624976000	0.373805000	0.376195000
0.124976000	0.873805000	0.876195000
0.124976000	0.873805000	0.376195000
0.624976000	0.873805000	0.876195000
0.000000000	0.997551000	0.252449000
0.500000000	0.997551000	0.252449000
0.000000000	0.997551000	0.752449000
0.500000000	0.497551000	0.252449000
0.500000000	0.497551000	0.752449000
0.500000000	0.997551000	0.752449000
0.750000000	0.997551000	0.002449000
0.250000000	0.497551000	0.502449000
0.250000000	0.997551000	0.002449000
0.250000000	0.997551000	0.502449000
0.750000000	0.497551000	0.002449000
0.750000000	0.497551000	0.502449000
0.000000000	0.497551000	0.752449000
0.250000000	0.497551000	0.002449000
0.000000000	0.497551000	0.252449000
0.750000000	0.997551000	0.502449000
0.750000000	0.747551000	0.252449000
0.750000000	0.747551000	0.752449000
0.000000000	0.247551000	0.002449000
0.000000000	0.247551000	0.502449000
0.000000000	0.747551000	0.502449000
0.500000000	0.247551000	0.002449000
0.500000000	0.247551000	0.502449000
0.500000000	0.747551000	0.002449000
0.000000000	0.747551000	0.002449000
0.250000000	0.247551000	0.252449000
0.250000000	0.247551000	0.752449000
0.250000000	0.747551000	0.252449000
0.250000000	0.747551000	0.752449000
0.750000000	0.247551000	0.252449000
0.500000000	0.747551000	0.502449000
0.750000000	0.247551000	0.752449000
0.375552000	0.750889000	0.249111000
0.375552000	0.250889000	0.749111000
0.375552000	0.250889000	0.249111000
0.874448000	0.250889000	0.999111000
0.874448000	0.750889000	0.499111000
0.874448000	0.250889000	0.499111000

0.374448000	0.750889000	0.999111000
0.375552000	0.750889000	0.749111000
0.874448000	0.750889000	0.999111000
0.875552000	0.250889000	0.249111000
0.124448000	0.750889000	0.749111000
0.875552000	0.750889000	0.249111000
0.875552000	0.750889000	0.749111000
0.124448000	0.250889000	0.249111000
0.124448000	0.250889000	0.749111000
0.124448000	0.750889000	0.249111000
0.374448000	0.750889000	0.499111000
0.624448000	0.250889000	0.249111000
0.624448000	0.250889000	0.749111000
0.624448000	0.750889000	0.249111000
0.875552000	0.250889000	0.749111000
0.374448000	0.250889000	0.999111000
0.125552000	0.750889000	0.499111000
0.625552000	0.750889000	0.999111000
0.000000000	0.876174000	0.499291000
0.000000000	0.876174000	0.999291000
0.500000000	0.376174000	0.499291000
0.500000000	0.376174000	0.999291000
0.500000000	0.876174000	0.499291000
0.500000000	0.876174000	0.999291000
0.125552000	0.000889000	0.249111000
0.125552000	0.000889000	0.749111000
0.125552000	0.500889000	0.249111000
0.125552000	0.500889000	0.749111000
0.625552000	0.000889000	0.249111000
0.625552000	0.000889000	0.749111000
0.625552000	0.500889000	0.249111000
0.625552000	0.500889000	0.749111000
0.125552000	0.250889000	0.499111000
0.125552000	0.250889000	0.999111000
0.624448000	0.750889000	0.749111000
0.125552000	0.750889000	0.999111000
0.625552000	0.250889000	0.499111000
0.625552000	0.250889000	0.999111000
0.625552000	0.750889000	0.499111000
0.374448000	0.250889000	0.499111000
0.250000000	0.250749000	0.374957000
0.374448000	0.000889000	0.249111000
0.250000000	0.750749000	0.374957000
0.624448000	0.000889000	0.999111000
0.624448000	0.500889000	0.499111000
0.624448000	0.500889000	0.999111000
0.000000000	0.000709000	0.123826000
0.000000000	0.000709000	0.623826000
0.000000000	0.500709000	0.123826000
0.000000000	0.500709000	0.623826000
0.500000000	0.000709000	0.123826000
0.500000000	0.000709000	0.623826000
0.624448000	0.000889000	0.499111000
0.500000000	0.500709000	0.123826000
0.250000000	0.375043000	0.499251000
0.250000000	0.375043000	0.999251000
0.250000000	0.875043000	0.499251000

0.250000000	0.875043000	0.999251000
0.750000000	0.375043000	0.499251000
0.750000000	0.375043000	0.999251000
0.750000000	0.875043000	0.499251000
0.750000000	0.875043000	0.999251000
0.000000000	0.376174000	0.999291000
0.500000000	0.500709000	0.623826000
0.124448000	0.500889000	0.999111000
0.124448000	0.500889000	0.499111000
0.124448000	0.000889000	0.999111000
0.250000000	0.750749000	0.874957000
0.750000000	0.250749000	0.374957000
0.750000000	0.250749000	0.874957000
0.750000000	0.750749000	0.374957000
0.750000000	0.750749000	0.874957000
0.374448000	0.000889000	0.749111000
0.374448000	0.500889000	0.249111000
0.374448000	0.500889000	0.749111000
0.874448000	0.000889000	0.249111000
0.874448000	0.000889000	0.749111000
0.874448000	0.500889000	0.249111000
0.874448000	0.500889000	0.749111000
0.375552000	0.000889000	0.499111000
0.375552000	0.000889000	0.999111000
0.375552000	0.500889000	0.499111000
0.375552000	0.500889000	0.999111000
0.875552000	0.000889000	0.499111000
0.875552000	0.000889000	0.999111000
0.875552000	0.500889000	0.499111000
0.875552000	0.500889000	0.999111000
0.124448000	0.000889000	0.499111000
0.250000000	0.250749000	0.874957000
0.000000000	0.376174000	0.499291000
0.750000000	0.750709000	0.623826000
0.750000000	0.500749000	0.124957000
0.000000000	0.375043000	0.749251000
0.000000000	0.875043000	0.249251000
0.000000000	0.875043000	0.749251000
0.500000000	0.375043000	0.249251000
0.500000000	0.375043000	0.749251000
0.500000000	0.875043000	0.249251000
0.750000000	0.500749000	0.624957000
0.000000000	0.125043000	0.499251000
0.000000000	0.125043000	0.999251000
0.000000000	0.375043000	0.249251000
0.000000000	0.625043000	0.499251000
0.500000000	0.125043000	0.499251000
0.500000000	0.125043000	0.999251000
0.500000000	0.625043000	0.499251000
0.500000000	0.625043000	0.999251000
0.000000000	0.000749000	0.374957000
0.000000000	0.000749000	0.874957000
0.000000000	0.500749000	0.374957000
0.000000000	0.500749000	0.874957000
0.500000000	0.000749000	0.374957000
0.000000000	0.625043000	0.999251000
0.500000000	0.750749000	0.624957000

0.500000000	0.750749000	0.124957000
0.500000000	0.250749000	0.624957000
0.250000000	0.000709000	0.373826000
0.250000000	0.000709000	0.873826000
0.250000000	0.500709000	0.373826000
0.250000000	0.500709000	0.873826000
0.750000000	0.000709000	0.373826000
0.750000000	0.000709000	0.873826000
0.750000000	0.500709000	0.373826000
0.750000000	0.500709000	0.873826000
0.250000000	0.125043000	0.249251000
0.250000000	0.125043000	0.749251000
0.250000000	0.625043000	0.249251000
0.250000000	0.625043000	0.749251000
0.750000000	0.125043000	0.249251000
0.750000000	0.125043000	0.749251000
0.750000000	0.625043000	0.249251000
0.750000000	0.625043000	0.749251000
0.000000000	0.250749000	0.124957000
0.000000000	0.250749000	0.624957000
0.000000000	0.750749000	0.124957000
0.000000000	0.750749000	0.624957000
0.500000000	0.250749000	0.124957000
0.500000000	0.000749000	0.874957000
0.500000000	0.500749000	0.374957000
0.500000000	0.875043000	0.749251000
0.250000000	0.126174000	0.499291000
0.000000000	0.750709000	0.373826000
0.000000000	0.750709000	0.873826000
0.500000000	0.250709000	0.373826000
0.500000000	0.250709000	0.873826000
0.500000000	0.750709000	0.373826000
0.500000000	0.750709000	0.873826000
0.250000000	0.376174000	0.249291000
0.250000000	0.376174000	0.749291000
0.250000000	0.876174000	0.249291000
0.000000000	0.250709000	0.873826000
0.250000000	0.876174000	0.749291000
0.750000000	0.376174000	0.749291000
0.500000000	0.500749000	0.874957000
0.750000000	0.876174000	0.749291000
0.250000000	0.000749000	0.124957000
0.250000000	0.000749000	0.624957000
0.250000000	0.500749000	0.124957000
0.250000000	0.500749000	0.624957000
0.750000000	0.000749000	0.124957000
0.750000000	0.000749000	0.624957000
0.750000000	0.376174000	0.249291000
0.000000000	0.250709000	0.373826000
0.750000000	0.876174000	0.249291000
0.500000000	0.626174000	0.249291000
0.500000000	0.626174000	0.749291000
0.250000000	0.126174000	0.999291000
0.250000000	0.626174000	0.499291000
0.250000000	0.626174000	0.999291000
0.750000000	0.126174000	0.999291000
0.750000000	0.626174000	0.499291000

0.750000000	0.626174000	0.999291000
0.250000000	0.250709000	0.123826000
0.250000000	0.250709000	0.623826000
0.250000000	0.750709000	0.123826000
0.750000000	0.126174000	0.499291000
0.750000000	0.250709000	0.123826000
0.750000000	0.250709000	0.623826000
0.750000000	0.750709000	0.123826000
0.000000000	0.126174000	0.249291000
0.000000000	0.126174000	0.749291000
0.000000000	0.626174000	0.249291000
0.000000000	0.626174000	0.749291000
0.500000000	0.126174000	0.249291000
0.250000000	0.750709000	0.623826000
0.500000000	0.126174000	0.749291000
0.250000000	0.497636000	0.752364000
0.250000000	0.997636000	0.252364000
0.250000000	0.997636000	0.752364000
0.750000000	0.497636000	0.252364000
0.750000000	0.497636000	0.752364000
0.750000000	0.997636000	0.252364000
0.000000000	0.997636000	0.002364000
0.000000000	0.497636000	0.002364000
0.000000000	0.497636000	0.502364000
0.000000000	0.997636000	0.502364000
0.500000000	0.497636000	0.002364000
0.250000000	0.497636000	0.252364000
0.500000000	0.497636000	0.502364000
0.750000000	0.997636000	0.752364000
0.500000000	0.747636000	0.752364000
0.750000000	0.247636000	0.002364000
0.500000000	0.247636000	0.752364000
0.500000000	0.247636000	0.252364000
0.000000000	0.747636000	0.752364000
0.000000000	0.747636000	0.252364000
0.000000000	0.247636000	0.752364000
0.000000000	0.247636000	0.252364000
0.750000000	0.747636000	0.502364000
0.750000000	0.747636000	0.002364000
0.750000000	0.247636000	0.502364000
0.250000000	0.747636000	0.502364000
0.250000000	0.747636000	0.002364000
0.250000000	0.247636000	0.502364000
0.250000000	0.247636000	0.002364000
0.500000000	0.997636000	0.002364000
0.500000000	0.747636000	0.252364000
0.500000000	0.997636000	0.502364000

==

Appendix S2. POSCAR file of the 4×4×4 supercell of KTN with only the oxygen vacancy

==

```
Vo-KTN
1.0
        15.9399995804         0.0000000000         0.0000000000
         0.0000000000        15.9695816040         0.0000000000
         0.0000000000         0.0000000000        15.9695816040
    K    Nb     O    Ta
   64    32   191    32
Direct
     0.375406000         0.373620000         0.126644000
     0.377045000         0.374345000         0.628412000
     0.375158000         0.873644000         0.127131000
     0.376534000         0.874245000         0.628271000
     0.874907000         0.373597000         0.126780000
     0.874815000         0.374066000         0.626897000
     0.124594000         0.373620000         0.126644000
     0.874891000         0.873708000         0.876935000
     0.874911000         0.623698000         0.876844000
     0.123466000         0.874245000         0.628271000
     0.625093000         0.373597000         0.126780000
     0.625185000         0.374066000         0.626897000
     0.625141000         0.873678000         0.126924000
     0.625100000         0.874064000         0.626843000
     0.122955000         0.374345000         0.628412000
     0.124842000         0.873644000         0.127131000
     0.121768000         0.374041000         0.373414000
     0.124922000         0.373687000         0.876721000
     0.625109000         0.873708000         0.876935000
     0.123418000         0.874031000         0.375587000
     0.124919000         0.873755000         0.876993000
     0.625324000         0.373964000         0.376469000
     0.625104000         0.874029000         0.377057000
     0.625020000         0.373620000         0.876658000
     0.378232000         0.374040000         0.373415000
     0.376582000         0.874031000         0.375587000
     0.375081000         0.873755000         0.876993000
     0.874676000         0.373964000         0.376469000
     0.874980000         0.373620000         0.876658000
     0.375078000         0.373687000         0.876721000
     0.874896000         0.874029000         0.377057000
     0.122999000         0.123143000         0.374804000
     0.874900000         0.874064000         0.626843000
     0.374978000         0.123846000         0.876654000
     0.377311000         0.625235000         0.374818000
     0.375048000         0.623638000         0.876762000
     0.875065000         0.123967000         0.376882000
     0.874895000         0.123660000         0.876799000
     0.875055000         0.624008000         0.376921000
     0.125022000         0.123846000         0.876654000
     0.122689000         0.625235000         0.374818000
     0.377001000         0.123143000         0.374804000
     0.874859000         0.873678000         0.126924000
```

0.625105000	0.123660000	0.876799000
0.624945000	0.624008000	0.376921000
0.625089000	0.623698000	0.876844000
0.124899000	0.123689000	0.127071000
0.123275000	0.123288000	0.628212000
0.124952000	0.623638000	0.876762000
0.624935000	0.123967000	0.376882000
0.124889000	0.623514000	0.127226000
0.123098000	0.625287000	0.628431000
0.376725000	0.123288000	0.628212000
0.625153000	0.123603000	0.126839000
0.624958000	0.124033000	0.626782000
0.625134000	0.623738000	0.126888000
0.375101000	0.123689000	0.127071000
0.624942000	0.624043000	0.626838000
0.375111000	0.623514000	0.127226000
0.874847000	0.123603000	0.126839000
0.875042000	0.124033000	0.626782000
0.874866000	0.623738000	0.126888000
0.875058000	0.624044000	0.626838000
0.376902000	0.625288000	0.628431000
0.000000000	0.246599000	0.003892000
0.500070000	0.746669000	0.004196000
0.500617000	0.248824000	0.503704000
0.000000000	0.748279000	0.504085000
0.500087000	0.246599000	0.003892000
0.500218000	0.748279000	0.504085000
0.000000000	0.746669000	0.004196000
0.250000000	0.248368000	0.753470000
0.250000000	0.747480000	0.254440000
0.250000000	0.747772000	0.754580000
0.750000000	0.247264000	0.253950000
0.750000000	0.747264000	0.754232000
0.750000000	0.247194000	0.753992000
0.000000000	0.248824000	0.503704000
0.750000000	0.747400000	0.254254000
0.250000000	0.248178000	0.253093000
0.000000000	0.497619000	0.253710000
0.250000000	0.513048000	0.502120000
0.000000000	0.997422000	0.253821000
0.500286000	0.997377000	0.754325000
0.500466000	0.497619000	0.253710000
0.500169000	0.497459000	0.754133000
0.750000000	0.996659000	0.004138000
0.250000000	0.496887000	0.004118000
0.500378000	0.997422000	0.253821000
0.250000000	0.994910000	0.504015000
0.750000000	0.496716000	0.004119000
0.750000000	0.498308000	0.504028000
0.000000000	0.497459000	0.754133000
0.750000000	0.998260000	0.504105000
0.000000000	0.997377000	0.754325000
0.250000000	0.996864000	0.004464000
0.001025000	0.250848000	0.624243000
0.250000000	0.500310000	0.873075000
0.750000000	0.000914000	0.373687000
0.750000000	0.001070000	0.873604000

0.750000000	0.500921000	0.373756000
0.750000000	0.501086000	0.873531000
0.250000000	0.125886000	0.252605000
0.250000000	0.623426000	0.252632000
0.750000000	0.124925000	0.248707000
0.000264000	0.750652000	0.624527000
0.750000000	0.624868000	0.248654000
0.750000000	0.624982000	0.748520000
0.000483000	0.250943000	0.124647000
0.250000000	0.001525000	0.873629000
0.000202000	0.751004000	0.124679000
0.250000000	0.491121000	0.377090000
0.499517000	0.250943000	0.124647000
0.250000000	0.624202000	0.745579000
0.250000000	0.001039000	0.374535000
0.750000000	0.125066000	0.748547000
0.250000000	0.125389000	0.745600000
0.499855000	0.000915000	0.374607000
0.499798000	0.751004000	0.124679000
0.499841000	0.375066000	0.748641000
0.499713000	0.874808000	0.248647000
0.750000000	0.500979000	0.624368000
0.003266000	0.125662000	0.498448000
0.000266000	0.125164000	0.998601000
0.750000000	0.374710000	0.498410000
0.003085000	0.623759000	0.498454000
0.496734000	0.125662000	0.498448000
0.000126000	0.874960000	0.748573000
0.499734000	0.125164000	0.998601000
0.499736000	0.625130000	0.998622000
0.000145000	0.000915000	0.374607000
0.499813000	0.001012000	0.874758000
0.000730000	0.500951000	0.374645000
0.000553000	0.501153000	0.874618000
0.499270000	0.500951000	0.374645000
0.000264000	0.625130000	0.998622000
0.499736000	0.750652000	0.624527000
0.498975000	0.250848000	0.624243000
0.496915000	0.623759000	0.498454000
0.000287000	0.874808000	0.248647000
0.000187000	0.001012000	0.874758000
0.499818000	0.751104000	0.873767000
0.250000000	0.875761000	0.250098000
0.250000000	0.627641000	0.499421000
0.750000000	0.126338000	0.998559000
0.750000000	0.625685000	0.498461000
0.250000000	0.250557000	0.124543000
0.250000000	0.258262000	0.619753000
0.250000000	0.749455000	0.124496000
0.750000000	0.126255000	0.498461000
0.250000000	0.126303000	0.998921000
0.750000000	0.251094000	0.123560000
0.750000000	0.626236000	0.998527000
0.250000000	0.625904000	0.998871000
0.000097000	0.126062000	0.248488000
0.000074000	0.626075000	0.248484000
0.499903000	0.126062000	0.248488000

0.250000000	0.750688000	0.622978000
0.499987000	0.126189000	0.748589000
0.000159000	0.375066000	0.748641000
0.750000000	0.250944000	0.623368000
0.750000000	0.751030000	0.123595000
0.499997000	0.626220000	0.748624000
0.499995000	0.374950000	0.248512000
0.000013000	0.126189000	0.748589000
0.750000000	0.876078000	0.248673000
0.499926000	0.626075000	0.248484000
0.250000000	0.000000000	0.623915000
0.499404000	0.250702000	0.373533000
0.499514000	0.251017000	0.873569000
0.499816000	0.750677000	0.373602000
0.250000000	0.375786000	0.247697000
0.250000000	0.375913000	0.749652000
0.000486000	0.251017000	0.873569000
0.250000000	0.875950000	0.748174000
0.750000000	0.376271000	0.748488000
0.000182000	0.751104000	0.873767000
0.499447000	0.501153000	0.874618000
0.250000000	0.001782000	0.125473000
0.250000000	0.123367000	0.499838000
0.250000000	0.500008000	0.125749000
0.250000000	0.492386000	0.620747000
0.750000000	0.000977000	0.124614000
0.750000000	0.000954000	0.624540000
0.750000000	0.376195000	0.248633000
0.000596000	0.250702000	0.373533000
0.499874000	0.874960000	0.748573000
0.750000000	0.876189000	0.748511000
0.000184000	0.750677000	0.373602000
0.000005000	0.374950000	0.248512000
0.750000000	0.500981000	0.124624000
0.000212000	0.376348000	0.998555000
0.000000000	0.375895000	0.498186000
0.499249000	0.875920000	0.498641000
0.499774000	0.876254000	0.998630000
0.125868000	0.000898000	0.248446000
0.750000000	0.750862000	0.623532000
0.126008000	0.500688000	0.249371000
0.125900000	0.501018000	0.747493000
0.000003000	0.626220000	0.748624000
0.625564000	0.000798000	0.248217000
0.625536000	0.500795000	0.248422000
0.499788000	0.376348000	0.998555000
0.625445000	0.500882000	0.747829000
0.125914000	0.251423000	0.998451000
0.624326000	0.750951000	0.748236000
0.370815000	0.257486000	0.497622000
0.624831000	0.250302000	0.498648000
0.625466000	0.251419000	0.998287000
0.625185000	0.749425000	0.498324000
0.625537000	0.001025000	0.748187000
0.625500000	0.751211000	0.998355000
0.374086000	0.251423000	0.998451000
0.129185000	0.257486000	0.497622000

0.500478000	0.375895000	0.498186000
0.126507000	0.750373000	0.498227000
0.125866000	0.751207000	0.998525000
0.875679000	0.250898000	0.248339000
0.375301000	0.250742000	0.249014000
0.874534000	0.251419000	0.998287000
0.874815000	0.749425000	0.498324000
0.875169000	0.250302000	0.498648000
0.374134000	0.751207000	0.998525000
0.375450000	0.750710000	0.748328000
0.874500000	0.751211000	0.998355000
0.124550000	0.750710000	0.748328000
0.624321000	0.250898000	0.248339000
0.875674000	0.750951000	0.748236000
0.124699000	0.250741000	0.249013000
0.124604000	0.250844000	0.747572000
0.124636000	0.750346000	0.248478000
0.373493000	0.750373000	0.498227000
0.375396000	0.250844000	0.747572000
0.624227000	0.251146000	0.747879000
0.624334000	0.750757000	0.248264000
0.875773000	0.251146000	0.747879000
0.875666000	0.750757000	0.248264000
0.375364000	0.750346000	0.248478000
0.250000000	0.259019000	0.378050000
0.374100000	0.501018000	0.747493000
0.000751000	0.875920000	0.498641000
0.623568000	0.500318000	0.498812000
0.750000000	0.751048000	0.874663000
0.374240000	0.001025000	0.748302000
0.373992000	0.500688000	0.249371000
0.874436000	0.000798000	0.248217000
0.874463000	0.001025000	0.748187000
0.874464000	0.500795000	0.248422000
0.750000000	0.250868000	0.374778000
0.874555000	0.500882000	0.747829000
0.375356000	0.001297000	0.998484000
0.750000000	0.750820000	0.374720000
0.374132000	0.000898000	0.248446000
0.876167000	0.001314000	0.498359000
0.250000000	0.749734000	0.375590000
0.876432000	0.500318000	0.498812000
0.875775000	0.501262000	0.998329000
0.125273000	0.000030000	0.498041000
0.250000000	0.250279000	0.874092000
0.374727000	0.000030000	0.498041000
0.250000000	0.749652000	0.874690000
0.125760000	0.001025000	0.748302000
0.375246000	0.501319000	0.998454000
0.750000000	0.251046000	0.874586000
0.875706000	0.001334000	0.998313000
0.127762000	0.493827000	0.497857000
0.372238000	0.493827000	0.497857000
0.624225000	0.501262000	0.998329000
0.000382000	0.001082000	0.623494000
0.000625000	0.501078000	0.123652000
0.499833000	0.001025000	0.123627000

0.499618000	0.001082000	0.623494000
0.623833000	0.001314000	0.498359000
0.000167000	0.001025000	0.123627000
0.624294000	0.001334000	0.998313000
0.250000000	0.375064000	0.998861000
0.001260000	0.500881000	0.623212000
0.750000000	0.375233000	0.998518000
0.750000000	0.874662000	0.498621000
0.750000000	0.875112000	0.998597000
0.498740000	0.500881000	0.623212000
0.499375000	0.501078000	0.123652000
0.124754000	0.501319000	0.998454000
0.000226000	0.876254000	0.998630000
0.250000000	0.874303000	0.498505000
0.250000000	0.874931000	0.000000000
0.124644000	0.001297000	0.998484000
0.500055000	0.996727000	0.003998000
0.500151000	0.247419000	0.753942000
0.250000000	0.246855000	0.003638000
0.250000000	0.236874000	0.501807000
0.250000000	0.754213000	0.504111000
0.750000000	0.248222000	0.503798000
0.000000000	0.747681000	0.253772000
0.750000000	0.748161000	0.504086000
0.000000000	0.247419000	0.753942000
0.000000000	0.747568000	0.754343000
0.500410000	0.247436000	0.253512000
0.750000000	0.746807000	0.004115000
0.500301000	0.747567000	0.754343000
0.250000000	0.746758000	0.004558000
0.000000000	0.247435000	0.253512000
0.000000000	0.498002000	0.503657000
0.000000000	0.996727000	0.003998000
0.500390000	0.747681000	0.253772000
0.750000000	0.997298000	0.754036000
0.250000000	0.497590000	0.753805000
0.250000000	0.997368000	0.254187000
0.750000000	0.497509000	0.253972000
0.750000000	0.997386000	0.254054000
0.250000000	0.997903000	0.754245000
0.000000000	0.496793000	0.003881000
0.000000000	0.998217000	0.503892000
0.500069000	0.496793000	0.003881000
0.250000000	0.497079000	0.253362000
0.500626000	0.498002000	0.503657000
0.750000000	0.246773000	0.003873000
0.750000000	0.497366000	0.754016000
0.500084000	0.998217000	0.503892000

==

Appendix S3. POSCAR file of the 4×4×4 supercell of Fe-doped KTN

```
==================================================================
Fe-KTN
1.0
        15.9399995804          0.0000000000          0.0000000000
         0.0000000000         15.9695816040          0.0000000000
         0.0000000000          0.0000000000         15.9695816040
   Fe     K    Nb     O    Ta
    1    64    31   191    32
Direct
     0.248133000         0.506900000         0.503556000
     0.374985000         0.872764000         0.627148000
     0.874023000         0.373725000         0.126531000
     0.874847000         0.374728000         0.625543000
     0.874251000         0.873455000         0.626221000
     0.125201000         0.375024000         0.625507000
     0.375863000         0.122322000         0.375174000
     0.873912000         0.873265000         0.126464000
     0.623887000         0.373770000         0.126580000
     0.623922000         0.374868000         0.625572000
     0.624061000         0.873237000         0.126479000
     0.624378000         0.873477000         0.626262000
     0.124043000         0.873237000         0.126430000
     0.124625000         0.374267000         0.876310000
     0.123530000         0.872920000         0.627002000
     0.874202000         0.373734000         0.876309000
     0.123141000         0.374388000         0.376768000
     0.375250000         0.374467000         0.376644000
     0.123206000         0.872795000         0.376136000
     0.124124000         0.873257000         0.876437000
     0.623996000         0.374709000         0.377636000
     0.623840000         0.373716000         0.876387000
     0.373745000         0.374957000         0.625865000
     0.624034000         0.873205000         0.876503000
     0.375059000         0.872714000         0.376164000
     0.373470000         0.374194000         0.876229000
     0.874148000         0.873468000         0.376998000
     0.373846000         0.873234000         0.876449000
     0.874564000         0.374627000         0.377646000
     0.624264000         0.873435000         0.377014000
     0.373896000         0.873154000         0.126522000
     0.873999000         0.873277000         0.876448000
     0.124291000         0.374274000         0.127423000
     0.373822000         0.123707000         0.876348000
     0.373386000         0.622737000         0.875937000
     0.874280000         0.123776000         0.376894000
     0.873998000         0.123556000         0.876479000
     0.874764000         0.622700000         0.377896000
     0.124151000         0.123776000         0.876282000
     0.124888000         0.622674000         0.378923000
     0.874138000         0.623122000         0.876353000
     0.623962000         0.123711000         0.376888000
     0.122651000         0.122356000         0.375121000
     0.623922000         0.622712000         0.377897000
     0.623923000         0.623202000         0.876359000
```

0.123966000	0.123612000	0.126592000
0.123119000	0.122525000	0.627352000
0.124585000	0.622736000	0.876034000
0.125989000	0.621549000	0.624431000
0.623997000	0.123570000	0.876500000
0.875011000	0.622590000	0.625413000
0.124381000	0.622841000	0.127630000
0.373489000	0.622896000	0.127706000
0.623958000	0.123525000	0.126556000
0.624061000	0.123772000	0.626360000
0.623912000	0.623291000	0.126526000
0.623872000	0.622606000	0.625445000
0.373716000	0.374173000	0.127503000
0.375700000	0.122527000	0.627522000
0.373092000	0.622794000	0.378996000
0.372417000	0.621918000	0.624521000
0.873917000	0.123563000	0.126475000
0.874450000	0.123888000	0.626296000
0.874047000	0.623282000	0.126440000
0.373941000	0.123617000	0.126694000
0.747289000	0.746186000	0.253799000
0.997280000	0.246575000	0.003554000
0.998426000	0.248314000	0.503593000
0.497184000	0.246520000	0.003707000
0.499033000	0.248416000	0.503685000
0.497216000	0.746165000	0.003649000
0.247269000	0.746332000	0.752775000
0.247380000	0.247382000	0.253025000
0.247449000	0.247424000	0.752752000
0.747179000	0.246601000	0.253837000
0.498278000	0.746837000	0.503877000
0.747311000	0.746115000	0.753471000
0.997347000	0.746198000	0.003577000
0.000000000	0.746890000	0.503871000
0.747248000	0.996402000	0.003584000
0.247344000	0.746121000	0.254138000
0.497612000	0.496600000	0.253847000
0.747329000	0.246571000	0.753600000
0.748159000	0.997374000	0.503700000
0.997144000	0.996302000	0.253333000
0.497634000	0.496373000	0.752785000
0.497631000	0.996168000	0.253403000
0.497617000	0.996238000	0.753766000
0.997283000	0.996310000	0.753646000
0.247327000	0.996609000	0.003482000
0.248279000	0.990413000	0.503407000
0.748765000	0.497123000	0.503713000
0.997955000	0.496280000	0.752739000
0.247581000	0.496697000	0.006694000
0.997628000	0.496552000	0.253594000
0.747417000	0.496413000	0.003310000
0.002380000	0.251111000	0.624483000
0.250523000	0.500204000	0.870143000
0.751283000	0.001480000	0.373382000
0.751385000	0.001512000	0.873513000
0.751173000	0.501299000	0.373228000
0.751021000	0.501415000	0.873665000

0.251479000	0.126535000	0.251963000
0.251364000	0.624040000	0.252468000
0.751226000	0.125564000	0.248517000
0.001214000	0.751472000	0.624803000
0.751271000	0.625286000	0.248475000
0.751383000	0.625338000	0.748584000
0.001920000	0.251435000	0.124699000
0.251338000	0.002306000	0.873622000
0.001508000	0.751309000	0.124553000
0.249685000	0.495605000	0.381625000
0.500895000	0.251652000	0.124718000
0.251345000	0.624611000	0.745257000
0.251422000	0.000797000	0.373954000
0.751378000	0.125539000	0.748588000
0.251552000	0.125932000	0.746349000
0.001647000	0.625325000	0.998591000
0.501167000	0.751236000	0.124613000
0.501104000	0.375309000	0.748421000
0.501111000	0.875267000	0.248559000
0.751334000	0.501274000	0.625252000
0.003455000	0.125914000	0.498308000
0.001620000	0.125510000	0.998652000
0.750758000	0.374371000	0.498409000
0.001605000	0.625152000	0.498661000
0.497494000	0.126307000	0.498413000
0.001542000	0.875434000	0.748705000
0.501210000	0.125548000	0.998670000
0.501148000	0.625248000	0.998580000
0.001263000	0.001453000	0.374314000
0.001542000	0.001478000	0.874685000
0.501353000	0.001487000	0.874721000
0.001829000	0.501332000	0.874512000
0.501135000	0.001553000	0.374421000
0.500352000	0.501290000	0.374886000
0.501449000	0.751344000	0.624721000
0.500142000	0.251427000	0.624400000
0.498842000	0.624836000	0.498593000
0.001620000	0.875315000	0.248458000
0.001310000	0.501253000	0.374397000
0.501286000	0.751282000	0.873551000
0.251339000	0.876389000	0.248627000
0.250355000	0.628184000	0.499394000
0.751382000	0.126594000	0.998563000
0.750768000	0.626390000	0.498554000
0.251009000	0.251539000	0.124643000
0.251238000	0.255722000	0.620157000
0.251407000	0.749884000	0.123777000
0.750330000	0.126626000	0.498320000
0.251507000	0.126926000	0.000000000
0.751287000	0.251431000	0.123535000
0.751357000	0.626424000	0.998550000
0.251477000	0.625835000	0.998459000
0.001477000	0.126552000	0.248399000
0.001582000	0.626319000	0.248559000
0.501211000	0.126601000	0.248535000
0.251241000	0.752678000	0.624096000
0.501425000	0.126575000	0.748670000

0.001779000	0.375170000	0.748541000
0.751177000	0.251370000	0.623453000
0.751309000	0.751394000	0.123415000
0.501268000	0.626311000	0.748450000
0.500913000	0.375561000	0.248759000
0.001459000	0.126511000	0.748729000
0.751316000	0.876537000	0.248506000
0.501130000	0.626245000	0.248675000
0.251446000	0.000000000	0.624398000
0.500576000	0.251395000	0.373527000
0.500863000	0.251562000	0.873547000
0.501275000	0.751270000	0.373486000
0.251568000	0.376638000	0.250400000
0.251616000	0.376009000	0.746874000
0.001870000	0.251385000	0.873498000
0.251442000	0.876569000	0.749702000
0.751371000	0.376382000	0.748655000
0.001490000	0.751359000	0.873550000
0.500078000	0.501312000	0.874348000
0.251330000	0.002382000	0.125077000
0.250137000	0.122163000	0.499577000
0.250488000	0.500309000	0.126178000
0.249826000	0.494813000	0.613934000
0.751333000	0.001512000	0.124536000
0.751281000	0.001638000	0.624656000
0.751266000	0.376462000	0.248456000
0.001940000	0.251072000	0.373419000
0.501359000	0.875381000	0.748587000
0.751387000	0.876593000	0.748582000
0.001260000	0.751303000	0.373357000
0.001617000	0.375332000	0.248597000
0.750928000	0.501412000	0.124427000
0.001591000	0.376407000	0.998603000
0.000630000	0.375228000	0.498253000
0.500895000	0.876839000	0.498609000
0.501278000	0.876489000	0.998650000
0.126737000	0.001739000	0.248568000
0.751227000	0.751474000	0.623649000
0.127011000	0.501383000	0.250773000
0.126639000	0.501433000	0.746312000
0.001661000	0.626389000	0.748521000
0.626359000	0.001571000	0.248410000
0.626050000	0.501411000	0.248615000
0.501131000	0.376517000	0.998611000
0.626251000	0.501424000	0.748838000
0.126794000	0.251745000	0.998660000
0.625348000	0.751479000	0.748552000
0.126708000	0.751397000	0.998509000
0.371568000	0.256432000	0.498117000
0.626314000	0.251608000	0.998480000
0.626284000	0.750728000	0.498351000
0.626446000	0.001599000	0.748421000
0.626402000	0.751452000	0.998500000
0.375138000	0.251736000	0.998624000
0.129747000	0.256177000	0.498083000
0.499928000	0.375685000	0.498255000
0.125567000	0.751921000	0.498486000

0.625320000	0.250402000	0.498899000
0.876435000	0.251641000	0.248707000
0.376222000	0.251696000	0.249218000
0.875486000	0.251595000	0.998511000
0.874967000	0.751050000	0.498316000
0.875634000	0.249874000	0.498914000
0.375284000	0.751431000	0.998491000
0.376477000	0.751291000	0.748532000
0.875467000	0.751471000	0.998470000
0.125521000	0.751360000	0.748560000
0.625114000	0.251817000	0.248684000
0.876531000	0.751483000	0.748565000
0.125799000	0.251673000	0.249427000
0.125667000	0.251404000	0.747721000
0.125652000	0.751144000	0.248387000
0.375465000	0.751710000	0.498534000
0.376378000	0.251384000	0.747813000
0.625200000	0.251686000	0.747985000
0.625205000	0.751412000	0.248317000
0.876624000	0.251613000	0.748059000
0.876475000	0.751412000	0.248330000
0.376310000	0.751179000	0.248510000
0.251148000	0.257224000	0.377917000
0.374926000	0.501553000	0.746048000
0.000000000	0.876938000	0.498492000
0.624962000	0.500337000	0.499317000
0.751348000	0.751426000	0.874594000
0.375468000	0.001614000	0.748614000
0.374475000	0.501549000	0.250986000
0.875352000	0.001589000	0.248301000
0.875424000	0.001606000	0.748442000
0.875352000	0.501383000	0.248295000
0.751120000	0.251416000	0.374662000
0.875423000	0.501405000	0.749190000
0.376430000	0.001571000	0.998534000
0.751114000	0.751439000	0.374419000
0.375311000	0.001703000	0.248422000
0.876374000	0.002316000	0.498527000
0.251160000	0.752140000	0.374650000
0.877615000	0.500345000	0.499204000
0.876590000	0.501410000	0.998601000
0.125661000	0.000000000	0.498236000
0.250995000	0.251104000	0.873998000
0.375654000	0.000335000	0.498224000
0.251413000	0.750026000	0.874424000
0.126656000	0.001635000	0.748488000
0.375904000	0.501551000	0.998277000
0.751314000	0.251432000	0.874552000
0.876553000	0.001596000	0.998471000
0.128096000	0.498124000	0.499557000
0.372104000	0.498256000	0.499722000
0.624902000	0.501425000	0.998563000
0.001473000	0.001555000	0.623564000
0.001855000	0.501369000	0.123466000
0.501321000	0.001457000	0.123515000
0.500986000	0.001687000	0.623522000
0.624634000	0.002309000	0.498505000

0.001547000	0.001435000	0.123470000
0.625327000	0.001636000	0.998436000
0.251595000	0.375291000	0.998664000
0.001116000	0.501030000	0.623975000
0.751348000	0.375336000	0.998534000
0.750367000	0.875776000	0.498541000
0.751388000	0.875452000	0.998561000
0.500584000	0.501117000	0.623567000
0.499953000	0.501359000	0.123642000
0.125711000	0.501465000	0.998373000
0.001592000	0.876536000	0.998608000
0.250614000	0.873299000	0.498577000
0.251395000	0.875309000	0.000000000
0.125622000	0.001634000	0.998547000
0.497389000	0.996422000	0.003450000
0.497421000	0.246680000	0.753603000
0.247415000	0.247087000	0.003182000
0.249329000	0.235246000	0.501677000
0.248570000	0.744198000	0.503701000
0.748618000	0.247633000	0.503657000
0.997274000	0.746277000	0.253626000
0.748848000	0.746878000	0.503650000
0.997533000	0.246711000	0.753398000
0.997382000	0.746102000	0.753459000
0.497546000	0.246621000	0.253566000
0.747376000	0.746409000	0.003440000
0.497406000	0.746091000	0.753503000
0.247431000	0.746201000	0.003321000
0.997107000	0.246700000	0.253457000
0.004285000	0.497286000	0.503309000
0.997354000	0.996500000	0.003360000
0.497515000	0.746211000	0.253715000
0.747512000	0.996478000	0.753485000
0.247927000	0.496729000	0.751677000
0.247570000	0.996150000	0.253494000
0.747711000	0.496540000	0.253580000
0.747453000	0.996481000	0.253540000
0.247576000	0.996687000	0.753257000
0.997801000	0.496501000	0.003042000
0.998319000	0.997333000	0.503454000
0.497312000	0.496524000	0.003238000
0.247784000	0.496579000	0.261054000
0.492855000	0.497458000	0.503306000
0.747273000	0.246625000	0.003586000
0.747860000	0.496426000	0.753094000
0.498180000	0.997118000	0.503506000

==

Appendix S4. POSCAR file of the 4×4×4 supercell of Mn-doped KTN

==

```
Mn-KTN
1.0
        15.9399995804         0.0000000000         0.0000000000
         0.0000000000        15.9695816040         0.0000000000
         0.0000000000         0.0000000000        15.9695816040
   Mn    K    Nb    O    Ta
    1   64    31  191    32
Direct
     0.249990000         0.503583000         0.502513000
     0.374479000         0.374595000         0.126391000
     0.374668000         0.374768000         0.626239000
     0.374862000         0.873536000         0.126243000
     0.375506000         0.873135000         0.626918000
     0.875055000         0.373932000         0.126272000
     0.875256000         0.374600000         0.625808000
     0.874998000         0.873593000         0.626163000
     0.125511000         0.374596000         0.126389000
     0.875027000         0.873562000         0.876229000
     0.125137000         0.873535000         0.126244000
     0.124485000         0.873136000         0.626917000
     0.624938000         0.373933000         0.126272000
     0.624738000         0.374601000         0.625810000
     0.624997000         0.873513000         0.126200000
     0.624996000         0.873594000         0.626162000
     0.125325000         0.374769000         0.626233000
     0.125712000         0.374556000         0.875814000
     0.124204000         0.374705000         0.375843000
     0.875094000         0.373948000         0.876197000
     0.875198000         0.374512000         0.376914000
     0.374832000         0.873639000         0.876217000
     0.374277000         0.374554000         0.875815000
     0.375791000         0.374704000         0.375838000
     0.375665000         0.873076000         0.375713000
     0.624968000         0.873504000         0.376428000
     0.624896000         0.373949000         0.876197000
     0.624799000         0.374511000         0.376914000
     0.125158000         0.873639000         0.876216000
     0.124335000         0.873075000         0.375713000
     0.624963000         0.873563000         0.876228000
     0.875032000         0.873505000         0.376432000
     0.875010000         0.623486000         0.876270000
     0.875001000         0.873514000         0.126197000
     0.374863000         0.123920000         0.876049000
     0.373748000         0.622081000         0.378028000
     0.374199000         0.622832000         0.875655000
     0.875223000         0.123758000         0.376335000
     0.875013000         0.123817000         0.876232000
     0.875467000         0.622753000         0.377248000
     0.124013000         0.123088000         0.375147000
     0.125129000         0.123918000         0.876049000
     0.375981000         0.123088000         0.375147000
     0.125790000         0.622833000         0.875653000
     0.624771000         0.123757000         0.376336000
```

0.624979000	0.123816000	0.876231000
0.624537000	0.622755000	0.377246000
0.624980000	0.623485000	0.876270000
0.125042000	0.123864000	0.126412000
0.124144000	0.123047000	0.627043000
0.126251000	0.622080000	0.378028000
0.126587000	0.621776000	0.625077000
0.624972000	0.123771000	0.126197000
0.624782000	0.123841000	0.626215000
0.624959000	0.623513000	0.126261000
0.624533000	0.622781000	0.625676000
0.374950000	0.123865000	0.126412000
0.375851000	0.123047000	0.627043000
0.374218000	0.622814000	0.126741000
0.125780000	0.622814000	0.126741000
0.373401000	0.621777000	0.625077000
0.875024000	0.123770000	0.126198000
0.875211000	0.123842000	0.626214000
0.875041000	0.623512000	0.126260000
0.875459000	0.622781000	0.625675000
0.499504000	0.746016000	0.503898000
0.749980000	0.246265000	0.253891000
0.249982000	0.746061000	0.753089000
0.250015000	0.745775000	0.254010000
0.249982000	0.247301000	0.753077000
0.249979000	0.247352000	0.252763000
0.000129000	0.745946000	0.003808000
0.499842000	0.745945000	0.003808000
0.500292000	0.247443000	0.503697000
0.499922000	0.246357000	0.003765000
0.000463000	0.746016000	0.503900000
0.999692000	0.247442000	0.503698000
0.000041000	0.246359000	0.003763000
0.749982000	0.745957000	0.753684000
0.750022000	0.745876000	0.253883000
0.749982000	0.246357000	0.753787000
0.000075000	0.496223000	0.253559000
0.749988000	0.996628000	0.503709000
0.999815000	0.996008000	0.253488000
0.999861000	0.996120000	0.753793000
0.499733000	0.496107000	0.753217000
0.500151000	0.996006000	0.253485000
0.500077000	0.996124000	0.753791000
0.499906000	0.496221000	0.253561000
0.249987000	0.996576000	0.003542000
0.249989000	0.990707000	0.503326000
0.749979000	0.496193000	0.003500000
0.749990000	0.496378000	0.503695000
0.000226000	0.496105000	0.753211000
0.249977000	0.496380000	0.002875000
0.749987000	0.996230000	0.003705000
0.250011000	0.125956000	0.745997000
0.250001000	0.494414000	0.378877000
0.250007000	0.500682000	0.871964000
0.750008000	0.001469000	0.373654000
0.750009000	0.001572000	0.873681000
0.750010000	0.501335000	0.373728000

0.750009000	0.501421000	0.874042000
0.250007000	0.126562000	0.252405000
0.250007000	0.624350000	0.251939000
0.001069000	0.251248000	0.624364000
0.750006000	0.125484000	0.248609000
0.750010000	0.125479000	0.748440000
0.750008000	0.625309000	0.248711000
0.750010000	0.625441000	0.748547000
0.000545000	0.251550000	0.124814000
0.250009000	0.002530000	0.873738000
0.000140000	0.751429000	0.124746000
0.000027000	0.751210000	0.624570000
0.499475000	0.251552000	0.124814000
0.250010000	0.625154000	0.747175000
0.250009000	0.001301000	0.374308000
0.000575000	0.501408000	0.374904000
0.499869000	0.751428000	0.124747000
0.000251000	0.875315000	0.248676000
0.000041000	0.875418000	0.748468000
0.499798000	0.375323000	0.248762000
0.499991000	0.375150000	0.748490000
0.499756000	0.875315000	0.248676000
0.750011000	0.501253000	0.625120000
0.003194000	0.126160000	0.498405000
0.000137000	0.125550000	0.998625000
0.000216000	0.375323000	0.248760000
0.002224000	0.624637000	0.498743000
0.496817000	0.126163000	0.498406000
0.499881000	0.125551000	0.998625000
0.497789000	0.624633000	0.498742000
0.499879000	0.625418000	0.998725000
0.000075000	0.001593000	0.374528000
0.000066000	0.001538000	0.874644000
0.499949000	0.001538000	0.874643000
0.999669000	0.501377000	0.874890000
0.499940000	0.001594000	0.374527000
0.000140000	0.625419000	0.998724000
0.499989000	0.751208000	0.624569000
0.498947000	0.251250000	0.624363000
0.499441000	0.501408000	0.374909000
0.499932000	0.751424000	0.873771000
0.250006000	0.123560000	0.499748000
0.250005000	0.627580000	0.499472000
0.250009000	0.626298000	0.998709000
0.750009000	0.126408000	0.998558000
0.750008000	0.626189000	0.498598000
0.750009000	0.626364000	0.998659000
0.250006000	0.250719000	0.124838000
0.250008000	0.257640000	0.620490000
0.250007000	0.750638000	0.124165000
0.750006000	0.126354000	0.498336000
0.250010000	0.126534000	0.999242000
0.750008000	0.251406000	0.123850000
0.750007000	0.751508000	0.123787000
0.000080000	0.126353000	0.248528000
0.999903000	0.126317000	0.748604000
0.000219000	0.626102000	0.248708000

0.000066000	0.626209000	0.748488000
0.499933000	0.126353000	0.248528000
0.250008000	0.752704000	0.624209000
0.500114000	0.126318000	0.748604000
0.000028000	0.375149000	0.748491000
0.750007000	0.251363000	0.623537000
0.499953000	0.626208000	0.748487000
0.499789000	0.626099000	0.248709000
0.750006000	0.876302000	0.248595000
0.000081000	0.751135000	0.373806000
0.000082000	0.751426000	0.873771000
0.499196000	0.251214000	0.373834000
0.499528000	0.251508000	0.873733000
0.499929000	0.751135000	0.373807000
0.250008000	0.376001000	0.248280000
0.250011000	0.375281000	0.750015000
0.250006000	0.876408000	0.248923000
0.000491000	0.251507000	0.873734000
0.250011000	0.876661000	0.749496000
0.750010000	0.376083000	0.748651000
0.500346000	0.501376000	0.874890000
0.750010000	0.876410000	0.748464000
0.250007000	0.002663000	0.125248000
0.250010000	0.000342000	0.624212000
0.250006000	0.500709000	0.125013000
0.250000000	0.494971000	0.621571000
0.750008000	0.001574000	0.124623000
0.750009000	0.001560000	0.624470000
0.750006000	0.376151000	0.248620000
0.000821000	0.251212000	0.373833000
0.499977000	0.875419000	0.748468000
0.750008000	0.501439000	0.124848000
0.750010000	0.374473000	0.498439000
0.999021000	0.375477000	0.498367000
0.000101000	0.876403000	0.998620000
0.500993000	0.375482000	0.498367000
0.499983000	0.376203000	0.998709000
0.500300000	0.876453000	0.498601000
0.499915000	0.876404000	0.998621000
0.125599000	0.001896000	0.248560000
0.750006000	0.751528000	0.623670000
0.125763000	0.501584000	0.249959000
0.125047000	0.501630000	0.748703000
0.999715000	0.876454000	0.498600000
0.625432000	0.001659000	0.248359000
0.625360000	0.501559000	0.248898000
0.625573000	0.501714000	0.749175000
0.128721000	0.258099000	0.498393000
0.125691000	0.251760000	0.998636000
0.624496000	0.751656000	0.748364000
0.125551000	0.751782000	0.998529000
0.624763000	0.250235000	0.498847000
0.625378000	0.251640000	0.998479000
0.625272000	0.750901000	0.498393000
0.625461000	0.001674000	0.748249000
0.625421000	0.751736000	0.998483000
0.125271000	0.752440000	0.498488000

0.374323000	0.251760000	0.998635000
0.375296000	0.751266000	0.248535000
0.375347000	0.251267000	0.747680000
0.375181000	0.251662000	0.249389000
0.874636000	0.251639000	0.998479000
0.874736000	0.750904000	0.498396000
0.875246000	0.250231000	0.498848000
0.374460000	0.751782000	0.998528000
0.375465000	0.751500000	0.748416000
0.874589000	0.751736000	0.998482000
0.875580000	0.251725000	0.248758000
0.124548000	0.751500000	0.748417000
0.875571000	0.751535000	0.248484000
0.875517000	0.751657000	0.748364000
0.124831000	0.251662000	0.249390000
0.124667000	0.251267000	0.747679000
0.124719000	0.751265000	0.248535000
0.374737000	0.752437000	0.498489000
0.624429000	0.251725000	0.248757000
0.624426000	0.251566000	0.747963000
0.624444000	0.751533000	0.248485000
0.875586000	0.251565000	0.747965000
0.371290000	0.258105000	0.498393000
0.250007000	0.258689000	0.377981000
0.125466000	0.001795000	0.748365000
0.250008000	0.752339000	0.374778000
0.750009000	0.251405000	0.874601000
0.750008000	0.751467000	0.374649000
0.750007000	0.751569000	0.874615000
0.374546000	0.001796000	0.748365000
0.374248000	0.501584000	0.249963000
0.374964000	0.501631000	0.748699000
0.874577000	0.001661000	0.248359000
0.874550000	0.001675000	0.748248000
0.874651000	0.501557000	0.248894000
0.750008000	0.251367000	0.374791000
0.874439000	0.501714000	0.749177000
0.375403000	0.001803000	0.998485000
0.374410000	0.001897000	0.248558000
0.375641000	0.501701000	0.998621000
0.875799000	0.002518000	0.498412000
0.875520000	0.001794000	0.998393000
0.876227000	0.500594000	0.499369000
0.875464000	0.501719000	0.999091000
0.125070000	0.000773000	0.498181000
0.250007000	0.250025000	0.874285000
0.374942000	0.000776000	0.498181000
0.250007000	0.751163000	0.874691000
0.371723000	0.494745000	0.500346000
0.128281000	0.494747000	0.500345000
0.124611000	0.001803000	0.998485000
0.624493000	0.001794000	0.998393000
0.623798000	0.500593000	0.499369000
0.624548000	0.501718000	0.999091000
0.000217000	0.001673000	0.623570000
0.000238000	0.501408000	0.123998000
0.000136000	0.501211000	0.624090000

0.499903000	0.001518000	0.123771000
0.499799000	0.001674000	0.623568000
0.624211000	0.002519000	0.498412000
0.000112000	0.001517000	0.123772000
0.250011000	0.374809000	0.998762000
0.250009000	0.873787000	0.498747000
0.250009000	0.875718000	0.999273000
0.750009000	0.375263000	0.998637000
0.750006000	0.875587000	0.498482000
0.750009000	0.875545000	0.998569000
0.000035000	0.376203000	0.998708000
0.499878000	0.501211000	0.624087000
0.499779000	0.501408000	0.124000000
0.124371000	0.501701000	0.998619000
0.500130000	0.246444000	0.253540000
0.000022000	0.745956000	0.753679000
0.000020000	0.745927000	0.253708000
0.000017000	0.246516000	0.753653000
0.999832000	0.246445000	0.253538000
0.749982000	0.746209000	0.503655000
0.249984000	0.746172000	0.003327000
0.749991000	0.246868000	0.503662000
0.249983000	0.743846000	0.503659000
0.249997000	0.237066000	0.501751000
0.249983000	0.246834000	0.003101000
0.499947000	0.246519000	0.753654000
0.499977000	0.996310000	0.003499000
0.749985000	0.746277000	0.003604000
0.749983000	0.246474000	0.003699000
0.749981000	0.496244000	0.753289000
0.749972000	0.996339000	0.753579000
0.495167000	0.496235000	0.503423000
0.249991000	0.496110000	0.257900000
0.499780000	0.496291000	0.003181000
0.000117000	0.996499000	0.503467000
0.004810000	0.496234000	0.503422000
0.000174000	0.496293000	0.003179000
0.999996000	0.996311000	0.003501000
0.749984000	0.996206000	0.253545000
0.749991000	0.496235000	0.253556000
0.249971000	0.996651000	0.753241000
0.249985000	0.996119000	0.253424000
0.249978000	0.496187000	0.747825000
0.500014000	0.745926000	0.253707000
0.499943000	0.745957000	0.753679000
0.499859000	0.996497000	0.503465000

==

Appendix S5. POSCAR file of the $\sqrt{2}\times\sqrt{2}\times 2$ supercell of orthorhombic KTN

===

```
O-phase-KTN
1.0
          5.6461000443        0.0000000000        0.0000000000
          0.0000000000        7.9699997902        0.0000000000
          0.0000000000        0.0000000000        5.6461000443
    K    Ta    Nb    O
    4     2     2    12
Direct
       0.000000000       0.749950000       0.507470000
       0.000000000       0.250050000       0.507470000
       0.500000000       0.249950000       0.007470000
       0.500000000       0.750050000       0.007470000
       0.000000000       0.000000000       0.015490000
       0.500000000       0.500000000       0.515490000
       0.000000000       0.500000000       0.015700000
       0.500000000       0.000000000       0.515700000
       0.000000000       0.248260000       0.994740000
       0.000000000       0.751740000       0.994740000
       0.500000000       0.748260000       0.494740000
       0.500000000       0.251740000       0.494740000
       0.247100000       0.000000000       0.242280000
       0.752900000       0.000000000       0.242280000
       0.747100000       0.500000000       0.742280000
       0.252900000       0.500000000       0.742280000
       0.750230000       0.000000000       0.745420000
       0.249770000       0.000000000       0.745420000
       0.250230000       0.500000000       0.245420000
       0.749770000       0.500000000       0.245420000
```

===

Appendix S6. POSCAR file of the $\sqrt{2}\times\sqrt{2}\times 2$ supercell of tetragonal KTN

==

```
T-phase-KTN
1.0
        5.6446876526         0.0000000000         0.0000000000
        0.0000000000         5.6446876526         0.0000000000
        0.0000000000         0.0000000000         8.0139999390
    K    Ta    Nb     O
    4     2     2    12
Direct
     0.000000000         0.500000000         0.255590000
     0.500000000         0.000000000         0.255590000
     0.500000000         0.000000000         0.755590000
     0.000000000         0.500000000         0.755590000
     0.000000000         0.000000000         0.010360000
     0.500000000         0.500000000         0.510360000
     0.000000000         0.000000000         0.510420000
     0.500000000         0.500000000         0.010420000
     0.000000000         0.000000000         0.244620000
     0.500000000         0.500000000         0.744620000
     0.000000000         0.000000000         0.747600000
     0.500000000         0.500000000         0.247600000
     0.748270000         0.748270000         0.497910000
     0.251730000         0.251730000         0.497910000
     0.251730000         0.748270000         0.497910000
     0.748270000         0.251730000         0.497910000
     0.248270000         0.248270000         0.997910000
     0.751730000         0.751730000         0.997910000
     0.751730000         0.248270000         0.997910000
     0.248270000         0.751730000         0.997910000
```
