# Supplementary material for: Deciphering the atomistic mechanism underlying highly tunable piezoelectric properties in perovskite ferroelectrics via transition metal doping
Source: Nat Commun. 2024 Dec 5;15:10619. doi: 10.1038/s41467-024-54842-6 (PMC11621366; doi:10.1038/s41467-024-54842-6)
Supplement: Supplementary file 2 — Reporting Summary [file 41467_2024_54842_MOESM2_ESM.pdf]

Corresponding author(s): Hao Tian, Fei Li, Shujun Zhang

Lasing Reporting Summary

Nature Research wishes to improve the reproducibility of the work that we publish. This form is intended for publication with all accepted papers reporting claims of lasing and provides structure for consistency and transparency in reporting. Some list items might not apply to an individual manuscript, but all fields must be completed for clarity.

For further information on Nature Research policies, including our data availability policy, see Authors & Referees.

Experimental design

Please check: are the following details reported in the manuscript?

1. Threshold

Plots of device output power versus pump power over a wide range of values indicating a clear threshold

- [ ] Yes
- [x] No

We used commercial laser sources for the Raman, S-E, and V-PFM measurements. The measurements in this work are not relevant to the lasing threshold.

2. Linewidth narrowing

Plots of spectral power density for the emission at pump powers below, around, and above the lasing threshold, indicating a clear linewidth narrowing at threshold

- [ ] Yes
- [x] No

We used commercial laser sources for the Raman, S-E, and V-PFM measurements. The measurements in this work are not relevant to the linewidth narrowing.

Resolution of the spectrometer used to make spectral measurements

- [x] Yes
- [ ] No

Included in the experimental description of Raman measurements in the manuscript.

3. Coherent emission

Measurements of the coherence and/or polarization of the emission

- [x] Yes
- [ ] No

Details of polarization of Raman measurements are included in the experimental description, while these of S-E and V-PFM measurements are not relevant. Coherence of the emission is not relevant to the measurements in this work.

4. Beam spatial profile

Image and/or measurement of the spatial shape and profile of the emission, showing a well-defined beam above threshold

- [ ] Yes
- [x] No

We used commercial laser sources for the Raman, S-E, and V-PFM measurements. The measurements in this work are not relevant to the beam spatial profile.

5. Operating conditions

Description of the laser and pumping conditions
Continuous-wave, pulsed, temperature of operation

- [ ] Yes
- [x] No

We used commercial laser sources for the Raman, S-E, and V-PFM measurements. The measurements in this work are not relevant to the laser and pumping conditions.

Threshold values provided as density values (e.g. W cm^{-2} or J cm^{-2}) taking into account the area of the device

- [ ] Yes
- [x] No

We used commercial laser sources for the Raman, S-E, and V-PFM measurements. The measurements in this work are not relevant to the threshold values.

6. Alternative explanations

Reasoning as to why alternative explanations have been ruled out as responsible for the emission characteristics
e.g. amplified spontaneous, directional scattering; modification of fluorescence spectrum by the cavity

- [ ] Yes
- [x] No

We used commercial laser sources for the Raman, S-E, and V-PFM measurements. The measurements in this work are not relevant to the information.

7. Theoretical analysis

Theoretical analysis that ensures that the experimental values measured are realistic and reasonable
e.g. laser threshold, linewidth, cavity gain-loss, efficiency

- [ ] Yes
- [x] No

We used commercial laser sources for the Raman, S-E, and V-PFM measurements. The theoretical analysis is not relevant, and not included in this work.

8. Statistics

Number of devices fabricated and tested

- [ ] Yes
- [x] No

We used commercial laser sources for the Raman, S-E, and V-PFM measurements. The work does not involve the fabrication and test of devices.

Statistical analysis of the device performance and lifetime (time to failure)

- [ ] Yes
- [x] No

We used commercial laser sources for the Raman, S-E, and V-PFM measurements. The statistical analysis is not relevant in this work.
